# Multi-monoubiquitylation controls VASP-mediated actin dynamics

**DOI:** 10.1242/jcs.261527

**Published:** 2024-01-29

**Authors:** Laura E. McCormick, Cristian Suarez, Laura E. Herring, Kevin S. Cannon, David R. Kovar, Nicholas G. Brown, Stephanie L. Gupton

**Affiliations:** ^1^ Department of Cell Biology and Physiology, University of North Carolina at Chapel Hill, Chapel Hill, NC 27599, USA; ^2^ Department of Molecular Genetics and Cell Biology, University of Chicago, Chicago, IL 60637, USA; ^3^ Department of Biochemistry and Molecular Biology, University of Chicago, Chicago, IL 60637, USA; ^4^ Michael Hooker Proteomics Core, University of North Carolina at Chapel Hill, Chapel Hill, NC 27599, USA; ^5^ Department of Pharmacology, University of North Carolina at Chapel Hill, Chapel Hill, NC 27599, USA; ^6^ Department of Biochemistry and Biophysics, University of North Carolina at Chapel Hill, Chapel Hill, NC 27599, USA; ^7^ Lineberger Comprehensive Cancer Center, University of North Carolina at Chapel Hill, Chapel Hill, NC 27599, USA; ^8^ Neuroscience Center, University of North Carolina at Chapel Hill, Chapel Hill, NC 27599, USA

**Keywords:** Actin, Ubiquitylation, VASP, TRIM9, Nondegradative, Filopodia

## Abstract

The actin cytoskeleton performs multiple cellular functions, and as such, actin polymerization must be tightly regulated. We previously demonstrated that reversible, non-degradative ubiquitylation regulates the function of the actin polymerase VASP in developing neurons. However, the underlying mechanism of how ubiquitylation impacts VASP activity was unknown. Here, we show that mimicking multi-monoubiquitylation of VASP at K240 and K286 negatively regulates VASP interactions with actin. Using *in vitro* biochemical assays, we demonstrate the reduced ability of multi-monoubiquitylated VASP to bind, bundle, and elongate actin filaments. However, multi-monoubiquitylated VASP maintained the ability to bind and protect barbed ends from capping protein. Finally, we demonstrate the electroporation of recombinant multi-monoubiquitylated VASP protein altered cell spreading morphology. Collectively, these results suggest a mechanism in which ubiquitylation controls VASP-mediated actin dynamics.

## Teaser

**Highlighted Article:** Two prominent monoubiquitylation sites on the actin polymerase VASP reduce the ability of VASP to bind, bundle and elongate actin filaments.

## INTRODUCTION

The dynamic remodeling of the actin cytoskeleton regulates many aspects of cell biology, including cell division, morphology, motility and endocytic trafficking. Starting from single monomers, actin polymerizes into filaments that interact with a myriad of actin regulatory proteins to form a variety of different cytoskeletal architectures (Pollard and Cooper, 2009). Local actin reorganization, polymerization and depolymerization are dependent upon spatiotemporal regulation by these different proteins.

Amidst the abundance of actin regulatory proteins, the Ena/VASP protein family [comprising Mena (encoded by *ENAH*), VASP and EVL] occupies a specialized niche (Krause et al., 2003; Bear and Gertler, 2009). The tetrameric Ena/VASP proteins bind and bundle parallel actin filaments. They also bind to actin monomers and the fast growing ‘barbed’ ends of actin filaments, where they enhance the addition of monomers and protect the barbed end from capping protein, which terminates actin polymerization (Faix and Rottner, 2022; Krause et al., 2003). The combination of these functions position Ena/VASP proteins as multi-faceted regulators of filopodia – finger-like bundled actin-rich protrusions that extend from the cell periphery. Although their precise function varies by cell type, filopodia are considered sensors that explore the local environment, influencing cell migration and morphology changes (Gupton and Gertler, 2010; Jacquemet et al., 2015). For example, developing murine cortical neurons require Ena/VASP proteins for filopodia formation and subsequent neurite initiation (Dent et al., 2007; Kwiatkowski et al., 2007). Impairment of Ena/VASP activity after neurite initiation disrupts growth cone filopodia formation and responses to guidance cues (Lebrand et al., 2004). Finally, VASP and EVL play a role in dendritic filopodia and dendritic spine formation (Lin et al., 2010; Parker et al., 2023).

Previously, we identified the brain-enriched E3 ubiquitin ligase TRIM9 as an interaction partner of VASP (Menon et al., 2015, 2021). VASP and TRIM9 colocalize at the tips of growth cone filopodia in developing neurons, suggesting that TRIM9 also regulates these structures. Loss of *Trim9* increases filopodia number and stability in the growth cone and impairs axon pathfinding. We also showed that TRIM9 is required for the non-degradative ubiquitylation of VASP in neurons (Menon et al., 2015). Interestingly, this was a reversible modification; addition of the guidance cue netrin-1 resulted in VASP deubiquitylation and a consequential increase in filopodia stability and number. Furthermore, experiments utilizing ubiquitin mutant constructs incapable of ubiquitin chain formation suggested that VASP was monoubiquitylated or multi-monoubiquitylated, as opposed to polyubiquitylated (Boyer et al., 2020). Although K48-linked ubiquitylation is associated with proteasomal degradation, monoubiquitylation is appreciated to regulate protein localization and activity (Baker et al., 2013; Lin et al., 2016; Nakagawa and Nakayama, 2015). As VASP ubiquitylation reduced filopodial stability, we hypothesized that monoubiquitylation or multi-monoubiquitylation negatively regulated VASP activity.

In this study, we investigate the mechanistic impact of ubiquitylation on VASP function. We identify two prominent ubiquitylation sites within VASP. Through biochemical reconstitutions, we demonstrate that mimicking multi-monoubiquitylation of VASP reduces actin filament side-binding and bundling. Furthermore, both monoubiquitylation and multi-monoubiquitylation diminish the ability of VASP to enhance actin filament elongation. Our results suggest that ubiquitylated VASP maintains barbed end association but is unable to efficiently add new actin monomers to the filament. In murine embryonic fibroblasts, electroporation of multi-monoubiquitylated VASP alters cell spreading morphology and decreases localization to the lamellipodia, filopodial tips and focal adhesions. Collectively, our findings mechanistically describe the negative regulation of VASP activity by reversible mono- and multi-monoubiquitylation.

## RESULTS

### Identification of ubiquitylated lysine residues on VASP

**Fig. 1.**
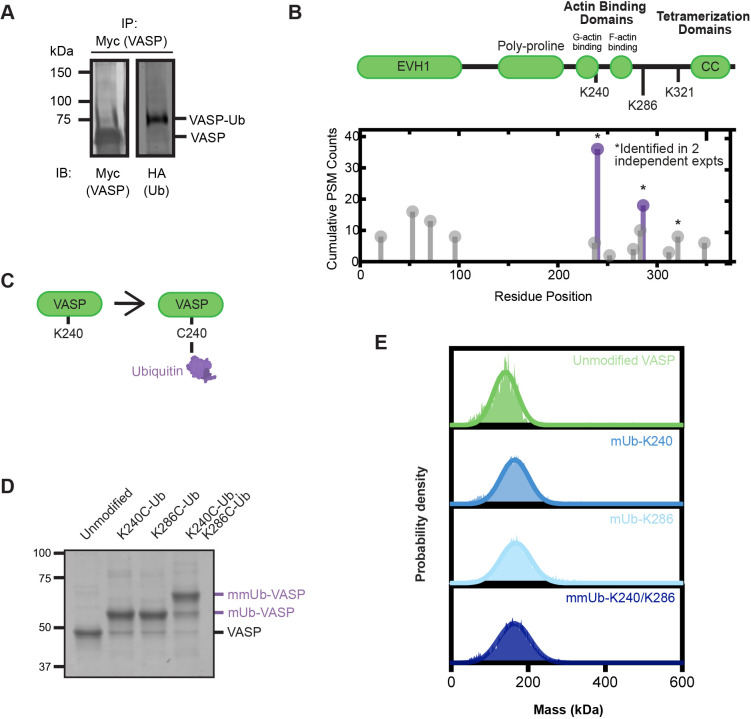
**VASP is ubiquitylated at K240 and K286.** (A) Western blot (IB) of ubiquitylated Myc–VASP. HEK293 cells were transfected with Myc–VASP and HA–ubiquitin, lysed and boiled in denaturing buffer before Myc immunoprecipitation (IP). Western blot is representative of two experimental repeats. (B) VASP domain architecture and proteomic detection of ubiquitylation sites on VASP by mass spectrometry. Purple residues were selected for follow-up study due to their abundance and replicability. Graph represents cumulative PSM counts from two experimental replicates. PSM, peptide-to-spectrum match. (C) Chemical ubiquitylation scheme, demonstrating the bismaleimidoethane-mediated crosslinking of ubiquitin G75C to cysteine-240 on VASP. (D) Coomassie-stained gel of purified, ubiquitylated VASP at indicated locations. Gel image is representative of two or three experimental replicates. (E) Mass photometry distributions of unmodified and ubiquitylated VASP. The estimated molecular mass was calculated with a Gaussian curve fit. These graphs show one representative trace for each construct (*n*=2).

We have previously demonstrated that non-degradative ubiquitylation of VASP impacted filopodial dynamics and axon guidance (Menon et al., 2015). Subsequent studies have indicated that VASP was likely monoubiquitylated or multi-monoubiquitylated (Boyer et al., 2020). However, the mechanistic consequence of VASP ubiquitylation – and how this impacts filopodial dynamics – was unknown. Consistent with neuronal experiments, VASP ubiquitylation in HEK293 cells is dependent upon the E3 ubiquitin ligase TRIM9 (Menon et al., 2015). To determine which residues on VASP were ubiquitylated, we immunoprecipitated exogenous Myc–VASP from HEK293 cells under denaturing conditions. Following separation by SDS-PAGE and immunoblotting, we observed two identifiable VASP bands – a prominent, unmodified VASP band at ∼65 kDa, and a higher molecular mass VASP band (∼75 kDa) that co-migrated with ubiquitin, indicating this was ubiquitylated VASP (VASP–Ub, Fig. 1A). This shift in molecular mass and relative ratio of unmodified and ubiquitylated VASP was similar to what we had previously observed with Myc–VASP in HEK293 cells and endogenous VASP in murine cortical neurons (Menon et al., 2015; Boyer et al., 2020).

Immunoprecipitated VASP was separated on an SDS-PAGE gel and excised for analysis by mass spectrometry. Following enzymatic digestion and analysis, we identified numerous lysine residues as ubiquitylation sites on VASP by unbiased mass spectrometry and confirmed these by targeted analysis (Fig. 1B). Despite multiple lysine residues identified, only three ubiquitylation sites were replicated across independent experiments and different enzymatic digestions. Of note, all of the additional ubiquitylation sites were identified in the same experimental replicate, corresponding to a higher level of overexpression of Myc–VASP (Fig. S1A). From these three sites, we selected the two most abundant ubiquitylation sites, K240 and K286, for investigation (Fig. S1B,C). K240 is located within the monomeric G-actin-binding (GAB) domain of VASP and K286 is located near to the filamentous actin-binding (FAB) domain, suggesting modification of these residues might influence actin binding.

### Generation of purified, ubiquitylated VASP mimics

To investigate how ubiquitylation at these sites alters protein function, we purified and ubiquitylated recombinant human VASP *in vitro*. Unlike phosphorylation or acetylation, modification of a protein with ubiquitin, an 8 kDa protein, cannot be mimicked with a single amino acid substitution. Instead, we utilized a chemical ubiquitylation approach in which a modified recombinant ubiquitin (ubiquitin^1-74, G75C^) was covalently conjugated to available cysteine residues on VASP (Fig. 1C). Three endogenous cysteines are present in human VASP, requiring their modification to specifically ubiquitylate residues of interest. Fortuitously, a cysteine-light version of VASP (VASP^CCC-SSA^) was previously generated and shown to have identical actin binding and elongation activity to unmodified VASP (Hansen and Mullins, 2010).

In neurons, we have observed that there are two discrete VASP bands that co-migrate with ubiquitin, suggesting multi-monoubiquitylation (Menon et al., 2015; Boyer et al., 2020). Studies utilizing ubiquitin mutants in HEK293 cells – identifying a single ubiquitylated VASP band – have confirmed VASP monoubiquitylation or multi-monoubiquitylation (Boyer et al., 2020). As phosphorylation of VASP at Ser157 causes a disproportionally large apparent molecular mass shift (∼4 kDa) (Smolenski et al., 1998), we cannot accurately interpret the apparent molecular mass shift observed for VASP–Ub by SDS-PAGE as monoubiquitylation or multi-monoubiquitylation. We created two VASP constructs with single cysteine mutations –VASP K240C and VASP K286C – within the cysteine-light VASP background construct to mimic two versions of monoubiquitylated VASP (mUb-K240 and mUb-K286). Based on the neuronal results suggesting multi-monoubiquitylation (Menon et al., 2015; Boyer et al., 2020), we also created a double cysteine mutant VASP K240C, K286C to mimic a multi-monoubiquitylated version of VASP protein (mmUb-K240/K286) (Fig. 1D). With each of these recombinant proteins, we achieved over 90% modification with ubiquitin after chemical crosslinking. Following successful chemical ubiquitylation, we used the bacterial sortase enzyme to conjugate a fluorophore-labeled peptide, TAMRA–PEG6–LPETGG, to a poly-glycine motif added at the N-terminus of VASP to fluorescently label the protein.

### VASP–Ub does not disrupt tetramerization

The C-terminal end of VASP contains an essential tetramerization domain (Fig. 1B). Studies manipulating oligomerization demonstrated Ena/VASP oligomer number directly correlated with the actin elongation rate *in vitro* (Brühmann et al., 2017) and filopodia formation in cultured cells (Harker et al., 2019). As VASP–Ub was associated with impaired filopodia number and stability in cultured neurons, we hypothesized ubiquitylation might impair VASP tetramerization and, consequently, VASP activity. We performed mass photometry to quantify the molecular mass of VASP. As a monomer of TAMRA–VASP is 41.3 kDa, the estimated tetrameric mass is 123.9 kDa. We detected a single peak corresponding to a tetramer for unmodified VASP centered ∼141 kDa. A similar single population was observed for mUb-K240 VASP, mUb-K286 VASP and mmUb-K240/K286 VASP (Fig. 1E). No significant peak shifts were observed beyond the margin of error. Thus, mUb-K240, mUb-K286 and mmUb-K240/K286 VASP each maintain tetramerization.

### mmUb-K240/K286 VASP exhibits decreased actin filament binding and bundling

**Fig. 2.**
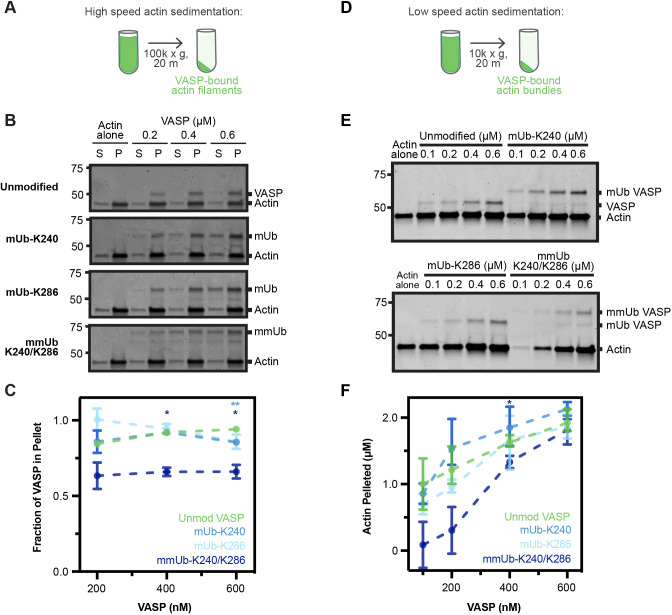
**Multi-monoubiquitylation of VASP impairs actin bundling and binding.** (A) High-speed actin co-sedimentation assay utilizing 1 µM actin and various concentrations of VASP. (B) Coomassie gels of supernatant and pellet fractions following centrifugation. (C) Quantification of VASP localized to the pellet fraction, determined by densitometry of Coomassie gels. Data points represent the mean±s.e.m. from three independent experiments. *P<0.0500; **P<0.0100 (two-way ANOVA with a Geisser-Greenhouse correction and the Dunnett's multiple comparisons test; unmodified VASP versus mUb-K240/K286: 400 nM=0.0315, 600 nM=0.0466; unmodified VASP versus mUb-K240: 600 nM=0.0089). (D) Low-speed actin co-sedimentation assay utilizing 2 µM actin and various concentrations of VASP. (E) Coomassie gels of the pellet fraction following centrifugation. (F) Quantification of actin localized to the pellet fraction, determined by densitometry of Coomassie gels. Data points represent the mean±s.e.m. from three independent experiments. *P<0.0500 (mixed effects model with a Geisser–Greenhouse correction and the Dunnett's multiple comparisons test; unmodified VASP versus mmUb-K240/K286: 200 nM=0.06399, 400 nM=0.0399).

With purified, ubiquitylated VASP tetramers in hand, we began testing the ability of VASP–Ub to bind actin filaments through high-speed co-sedimentation assays (Fig. 2A). At high centrifugation speeds, actin filaments – and any filamentous binding partners – are pelleted. Consistent with previous results (Reinhard et al., 1992; Breitsprecher et al., 2011), the majority of unmodified VASP co-sedimented with actin filaments (Fig. 2B,C). A single ubiquitylation modification at K286 (mUb-K286) did not significantly change the amount of VASP present in the pellet; however, a small but significant decrease was observed with 600 nM of mUb-K240. Furthermore, there was a significant decrease in the amount of mmUb-K240/K286 that co-sedimented with actin at 400 nM and 600 nM (Fig. 2C). These data suggest that VASP multi-monoubiquitylation decreases filamentous actin-binding affinity.

As VASP also promotes actin filament bundling, we completed similar assays at low centrifugation speeds to evaluate the ability of VASP to promote bundling of actin filaments (Fig. 2D). Compared to actin alone, unmodified VASP increased bundled actin in a concentration-dependent manner (Fig. 2E,F). We observed similar actin bundling activity in the presence of mUb-K240 and mUb-K286. A decrease in actin bundling activity was apparent for mmUb-K240/K286 at lower concentrations, although this change was only significant at 400 nM. By 600 nM – a concentration at which the actin bundling activity of unmodified VASP was saturated – the bundling activity of mmUb-K240/K286 recovered (Fig. 2E,F). Collectively, our data suggest a decreased ability of mmUb-K240/K286 VASP to both bind and bundle actin filaments.

### mmUb-K240/K286 VASP exhibits impaired ability to accelerate actin filament elongation

**Fig. 3.**
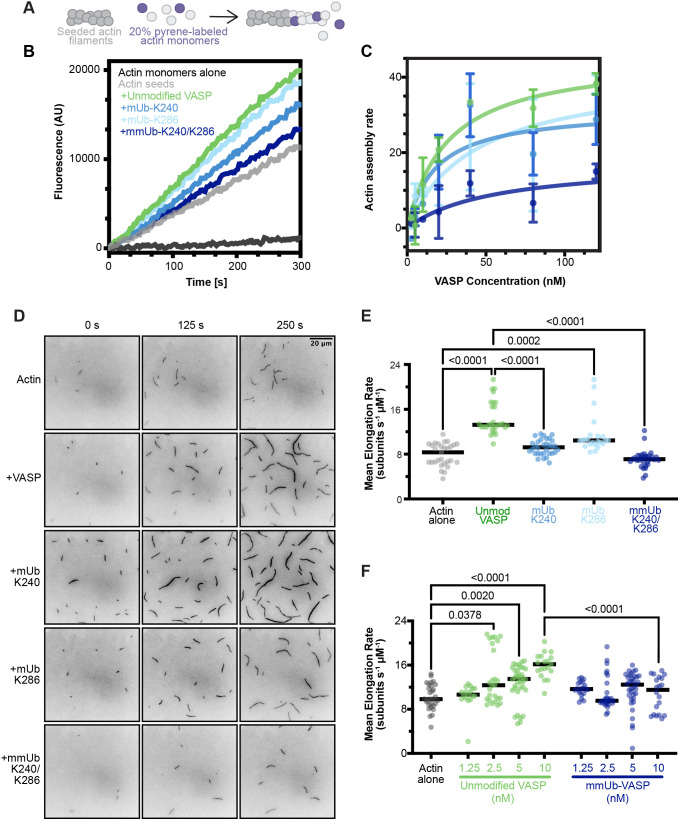
**Monoubiquitylation and multi-monoubiquitylation of VASP impairs elongation of actin filaments.** (A) Schematic of pyrene actin assays. (B) Fluorescent traces of 0.5 µM (20% pyrene labeled) actin monomer elongation from preformed actin seeds (0.5 µM) in the presence of 80 nM VASP. AU, arbitrary units. (C) The initial elongation rate from seeded pyrene elongation assays (calculated from the first 300 s of the assay) plotted against VASP concentration. A one-site binding curve was fit to each data set. The elongation rate of actin seeds alone was subtracted from each data point. Each data point represents the mean of 1–4 measurements collected across three independent experiments. Error bars represent standard deviation. (D) Polymerization of 1.5 µM actin (10% Alexa Fluor 488 labeled) visualized with TIRF microscopy for 10 min in the presence of 25 nM TAMRA–VASP. Inverted images in this panel only show actin at 0, 125 and 250 s of polymerization (full time-lapse imaging shown in Movie 1). (E) Quantification of actin filament elongation rates from the time-lapse images. *n*=23–32 filaments per condition from two or three experiments. Lines represent median elongation rate. Elongation rate was calculated assuming 375 subunits were added per µm. Statistical significance calculated with the Kruskal–Wallis test and Dunn's multiple comparison test. (F) Quantification of actin filament elongation rates at a range of VASP and mmUb-K240/K286 concentrations. *n*=17–36 filaments from two to four experiments for each data point. Lines represent median elongation rate. Indicated *P*-values were calculated with the Kruskal–Wallis test and Dunn's multiple comparison test.

The acceleration of actin filament elongation by VASP requires two separate actin-binding events. First, VASP must bind to the growing ‘barbed end’ of the actin filament through the FAB domain. Secondly, VASP binds monomeric actin through the GAB domain. This presence of four GAB domains – each able to bind an actin monomer – is thought to increase the local pool of monomeric actin available for polymerization at the barbed end, accelerating elongation (Hansen and Mullins, 2010). Owing to the proximity of the identified ubiquitylation sites to the GAB (K240-Ub) and the FAB (K286-Ub) domains, we hypothesized that ubiquitylated VASP might also exhibit a deficit in filament elongation. We used seeded pyrene assays – in which labeled actin monomers were added to pre-formed actin filaments – to specifically examine the elongation step of actin polymerization (Fig. 3A). We analyzed the first 300 s of the assay to determine the initial elongation rate at a range of VASP concentrations (Fig. 3B). Consistent with previous studies (Harker et al., 2019), unmodified VASP displayed a concentration-dependent increase in the rate of actin elongation before reaching a plateau.

mUb-K240 or mUb-K286 did not significantly change the plateaued maximal actin elongation rate. We also estimated similar *k*_D_^app^ values to those seen for unmodified VASP for both modifications (Table S1), suggesting the affinity of VASP for the barbed end of the actin filament was not detectably altered by either monoubiquitylation event. Strikingly, the cumulative effect of both monoubiquitins in mmUb-K240/K286 severely reduced the rate of actin elongation, with the values remaining close to that for actin alone. Although actin elongation was impaired by multi-monoubiquitylation, results from this bulk assay over multiple concentrations of mmUb-K240/K286 also suggested that the *k*_D_^app^ value was similar to that of unmodified VASP (Table S1). However, these calculations are limited by the uncertainty of the maximum elongation rate for this protein.

To examine changes in actin polymerase activity in greater detail, we utilized total internal reflection fluorescence microscopy (TIRFM) to determine the elongation rate of individual actin filaments. Each VASP construct (25 nM) was mixed with 1.5 µM actin monomers [10% Alexa Fluor (AF)488 labeled] and spontaneous actin assembly was imaged for 10 min (Fig. 3D; Fig. S2, Movie 1). Focusing on the elongation rates of single actin filaments and parallel actin bundles, quantification was largely consistent with bulk pyrene–actin polymerization. Unmodified VASP increased the mean actin elongation rate 1.8-fold compared to that with actin alone. Although the activity of mUb-K286 was similar to that of unmodified VASP, both mUb-K240 and mmUb-K240/K286 exhibited a decreased rate of elongation (Fig. 3D,E). Although we did not detect a significantly changed maximum elongation rate for mUb-K240 in the pyrene actin elongation assays, a large 95% confidence interval was calculated for these bulk measurements. In contrast, the ability to measure the elongation of single actin filaments in the TIRF assays might provide more accurate measurements.

As the time-lapse movies progressed, we observed VASP-mediated bundling of actin filaments. In the presence of unmodified VASP, actin bundles quickly predominated the field of view, obscuring the polymerization of single actin filaments (Fig. S2A). Actin filaments or bundles were traced to measure the mean intensity of actin along the structure to approximate the amount of bundling over time. We observed a significant increase in the mean fluorescence intensity of filaments with both unmodified VASP and mUb-K240 at 10 min. However, no significant change was observed for filament intensity with mUb-K286 and mmUb-K240/K286, suggesting decreased bundling activity (Fig. S2).

To further probe the striking difference in actin polymerase activity between unmodified and mmUb-K240/K286 VASP, we measured the actin elongation rate in the presence of either protein at a range of concentrations (1.25–10 nM). Although unmodified VASP exhibited a clear concentration-dependent increase in the elongation rate, mmUb-K240/K286 failed to significantly increase the rate of elongation compared to that for actin alone (Fig. 3E; Movie 2). Collectively, both seeded pyrene assays and TIRFM data support the conclusion that the enhancement of new actin monomer addition is impaired by multi-monoubiquitylation of VASP.

### Ubiquitylated VASP maintains anti-capping activity

Capping protein binds to the barbed ends of actin filaments and terminates actin polymerization (Cooper and Pollard, 1985). Previous work has demonstrated that VASP competes with capping protein for binding and thus protects the barbed end (Barzik et al., 2005). The anti-capping activity of Ena/VASP proteins is essential for their unique contribution to maintaining the actin cytoskeleton. In cultured cells, loss of VASP is associated with short, highly branched actin filaments (Bear et al., 2002). Previous work demonstrated that both GAB and FAB domain function is required for anti-capping activity (Barzik et al., 2005), suggesting that ubiquitylation at either K240 or K286 might modulate the anti-capping activity of VASP.

**Fig. 4.**
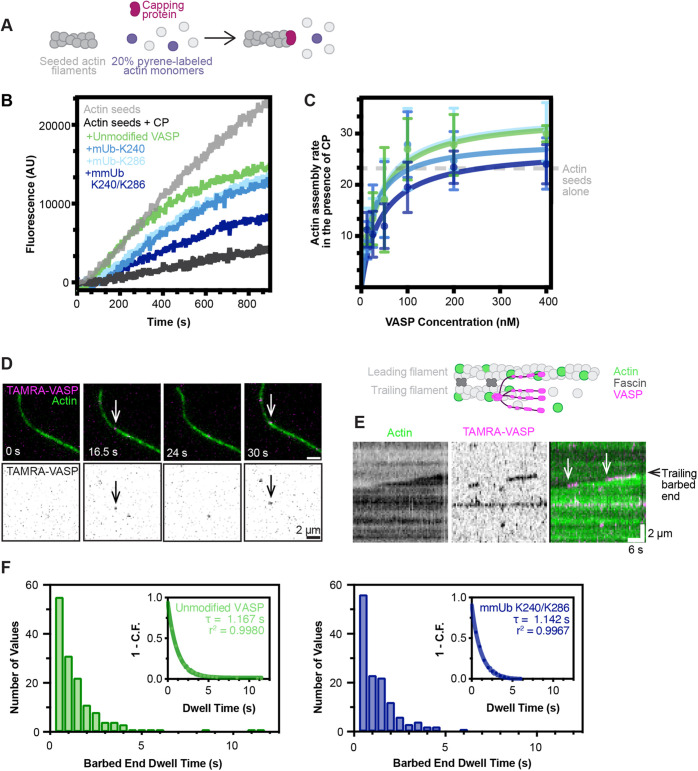
**Multi-monoubiquitylation of VASP does not change barbed end binding or anti-capping activity.** (A) Schematic of pyrene actin assays in the presence of capping protein. (B) Fluorescent traces of 0.5 µM (20% pyrene labeled) actin monomer elongation from preformed actin seeds (0.5 µM) in the presence of 5 nM capping protein (CP) and 50 nM VASP. AU, arbitrary units. (C) The initial elongation rate from seeded pyrene elongation assays in the presence of 5 nM CP (calculated from the first 300 s of the assay) plotted against VASP concentration. A one-site binding curve was fit to each data set. The dotted line represents the average elongation rate of actin seeds alone. Each data point represents the mean of 3–5 measurements collected across four independent experiments. Error bars represent standard deviation. (D) TIRFM demonstrating localization of 1 nM TAMRA–VASP to the barbed end of a trailing actin filament (1.5 µM actin, 10% Alexa Fluor 488 labeled) in a fascin-mediated actin bundle (673 nM fascin). Arrows indicate VASP bound to the trailing barbed end. (E) The growth of the trailing actin filament and the dynamic localization of VASP to the barbed end in D was visualized with kymograph analysis. Arrows denote two processive VASP binding events at the barbed end of two-filament bundles. These highly processive binding events are rare, with the majority of events lasting <1 s. (F) Frequency distribution of VASP barbed end binding events in D and E. Only VASP-binding events at the trailing barbed end of a two-filament bundle were quantified. Graph insets demonstrate [1−cumulative frequency (C.F.)] versus dwell time fit with a one-phase decay curve. *n*=145 events (unmodified VASP) and 131 events (mUb-K240/K286) from two experiments. **τ** represents time constant; r^2^, goodness of fit.

To evaluate the anti-capping activity of VASP-Ub, we performed seeded pyrene assays in the presence of 5 nM mammalian capping protein (Fig. 4A). Compared to what is seen with actin seeds alone, the addition of capping protein severely reduced actin elongation (Fig. 4B). As reported previously (Bear et al., 2002; Barzik et al., 2005), the addition of VASP counteracted capping protein in a concentration-dependent manner. All three ubiquitylated versions of VASP enhanced actin elongation over that seen with capping protein and actin (Fig. 4B,C), suggesting that monoubiquitylated and multi-monoubiquitylated VASP maintains anti-capping activity. As for the pyrene assays, in the absence of capping protein, our curve fitting calculated similar *k*_D_^app^ values, further supporting the hypothesis that unmodified and ubiquitylated VASP have a similar affinity of for the barbed end (Table S2).

N-terminal to the GAB domain, VASP also contains a poly-proline region that binds actin bound to a profilin family member (profilin–actin). Previously published *in vitro* experiments have mixed conclusions on the utilization of profilin–actin by Ena/VASP proteins, potentially due to differences in experimental design (Barzik et al., 2005; Hansen and Mullins, 2010; Breitsprecher et al., 2008; Brühmann et al., 2017). However, a recent cellular study suggested that Ena/VASP proteins do require profilin–actin for leading edge actin polymerization (Skruber et al., 2020). In our hands, we observed unmodified VASP (40 nM) was capable of utilizing profilin-1–actin. However, we did not observe an enhancement of actin elongation compared to what was seen with VASP plus actin alone (Fig. S3A–C). As seen above, mmUb-VASP failed to accelerate actin polymerization in the presence of actin alone or in the presence of profilin. Because mmUb-VASP demonstrated a decreased ability to accelerate elongation, whether mmUb-VASP could utilize profilin–actin was unclear. To circumvent this issue, we completed elongation assays in the presence of capping protein and profilin. We observed similar elongation rates for mmUb-VASP plus actin plus capping protein and mmUb-VASP plus actin plus capping protein plus profilin. As a result, we do not detect any deficit of mmUb-VASP to utilize profilin–actin (Fig. S3D–F).

Finally, to determine whether VASP ubiquitylation impairs actin monomer binding, we performed fluorescence polarization experiments with 40 nM AF488-labeled monomeric actin in low-salt conditions that discourage polymerization. With increasing concentrations of unmodified VASP, we observed a dose-dependent increase in fluorescence polarization of monomeric actin, indicative of VASP binding. We observed an overlapping dose-dependent increase in fluorescence polarization with increasing concentrations of mmUb-K240/K286, suggesting the affinity of mmUb-K240/K286 to bind monomeric actin is similar to unmodified VASP (Fig. S3G).

### mmUb-K240/K286 VASP maintains association with barbed end

We observed striking deficits in the ability of mmUb-K240/K286 to increase the elongation rate of actin filaments, yet the protein still possessed anti-capping activity. To determine whether VASP mmUb-K240/K286 maintained interaction with the barbed end of actin filaments, we quantified the lifetime of VASP puncta bound to the barbed end by single-molecule TIRFM imaging. This experiment is complicated by the relatively short lifetime of human VASP, previously measured at τ_1_=1.45 s (Hansen and Mullins, 2010) and τ_1_=1 s (Harker et al., 2019). However, the lifetime of VASP is increased on the trailing filament ends of fascin-bundled actin (τ_1_=2.6 s and 4.2 s for bundles containing two and three or more filaments, respectively) (Harker et al., 2019). Owing to the maximum acquisition speed of the microscope (0.5 s/ frame), we utilized fascin-bundled actin to more accurately quantify the lifetimes of unmodified VASP and VASP mmUb-K240/K286 on the trailing filament of a two-filament bundle (Fig. 4D,E). We estimated the lifetime of unmodified VASP at τ_1_=1.167 s (95% c.i.=1.094 to 1.245 s). We observed a similar dwell time (τ_1_=1.142 s; 95% c.i.=1.007 to 1.301 s) for mmUb-K240/K286 at the barbed end (Fig. 4F). As the inverse of puncta lifetime is a measurement of the *k*_off_ rate, we do not detect a change in between the k_off_ rate of unmodified VASP versus mmUb-VASP.

### mmUb-K240/K286 VASP exhibits impaired function and localization in cells

Previously, we have demonstrated that the mutation of lysine residues in VASP – reducing VASP ubiquitylation – resulted in increased filopodial number and stability in cultured murine neurons (Menon et al., 2015; Boyer et al., 2020). However, there is no primary coding sequence mutation that can mimic the ubiquitylation of VASP in cells. To examine how ubiquitylation of VASP altered its function and localization in cells, we electroporated purified ubiquitylated VASP into cultured cells. Given that Ena/VASP proteins can hetero-tetramerize (Riquelme et al., 2015), we utilized MV^D7^ cells (Bear et al., 2000), a mouse embryonic fibroblast line lacking all Ena/VASP family members, to simplify experiment interpretation. In this line, *Enah* (the gene encoding Mena) and *Vasp* were genetically deleted before subsequent selection for undetectable levels of EVL protein. Following electroporation of TAMRA-labeled VASP, cells were permitted to spread for 30 min before fixation.

**Fig. 5.**
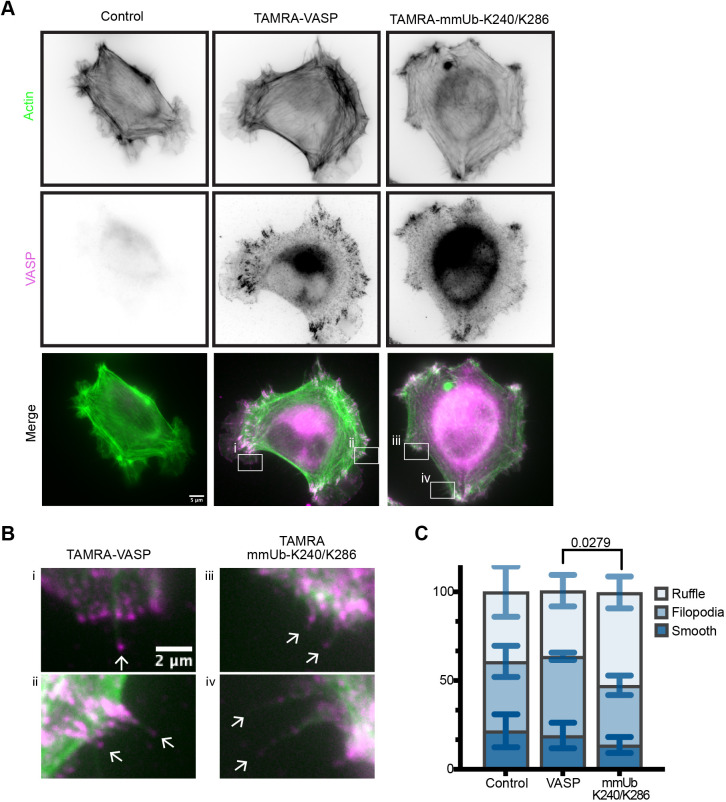
**Multi-monoubiquitylation of VASP enhances actin ruffling during cell spreading.** (A) Representative widefield images of fixed MV^D7^ cells with filopodial spreading morphology. Cells were electroporated with TAMRA–VASP (or buffer control) and allowed to spread for 30 min on fibronectin. (B) Magnified views of areas highlighted in A showing VASP localization to the tips of filopodia. (C) Classification of cell spreading into smooth-edged, filopodial or ruffled phenotypes. The plot indicates mean±s.d. percentage of each classification across three experiments. A Chi-square test for the goodness of fit was used to compare outcomes with a Bonferroni correction. 92–104 cells were classified across the experiments.

Previous work characterized spreading of MV^D7^ cells into three phenotypes – smooth-edged cells containing a clear lamellipodia, ruffled cells characterized by actin ruffles, and filopodial-rich cells (Applewhite et al., 2007) (Fig. 5A; Fig. S4A). Using a low concentration of TAMRA–VASP electroporated into cells, we observed no changes in the distribution of these three classes from cells that were electroporated with buffer alone. However, electroporation of mmUb-K240/K286 VASP decreased the proportion of filopodial-like cells and subsequently increased the proportion of ruffled cells (Fig. 5C). We observed no significant changes in filopodia density (in cells classified as filopodial), cell area or phalloidin staining following electroporation of either construct (Fig. S4B).

**Fig. 6.**
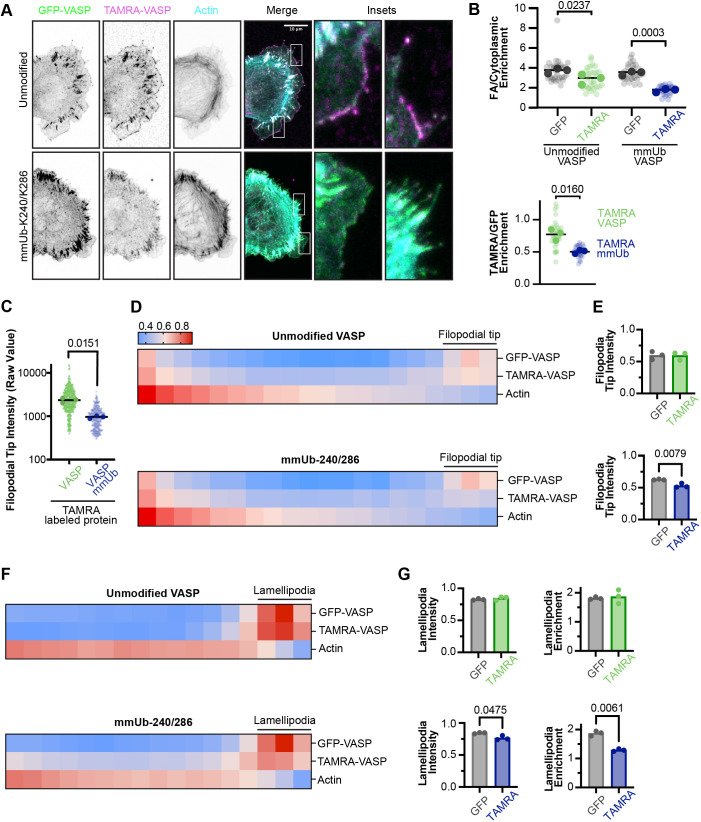
**Multi-monoubiquitylation of VASP reduces enrichment at both filopodial tips and lamellipodia.** (A) Representative maximum projection images of fixed MV^D7^ cells stably expressing GFP–VASP and electroporated with TAMRA–VASP. (B) Quantification of VASP enrichment at focal adhesions (FA) normalized to cytoplasmic intensity. Line represents the median VASP enrichment at focal adhesions. The enrichment of TAMRA–VASP or TAMRA–mmUb VASP at focal adhesions was then normalized to the GFP–VASP localization in each cell. Line represents the mean enrichment of TAMRA-labeled protein normalized to GFP–VASP. Statistics were calculated with a one-way ANOVA with Sidak's multiple comparisons or a paired two-tailed *t*-test, respectively. *n*=32 (unmodified) and 31 (mmUb-K240/K286 VASP) cells across three experiments for all focal adhesion measurements. (C) Raw intensity values of average filopodial tip intensity from linescans. The filopodial tip was defined as the first four pixels (0.255 µm) in a linescan drawn from tip to base. Each large data point represents the average from one independent experiment with smaller data points representing individual filopodia. The line represents the mean of three independent experiments. Statistics were calculated with a paired two-tailed *t*-test. *n*=34 (unmodified) and 35 (mmUb-K240/K286 VASP) cells across three experiments for all filopodia measurements. (D) Heatmap localization of proteins from throughout the length of the filopodia. The fluorescence intensity of each protein with a filopodium was normalized and the length binned. (E) Quantification of normalized VASP intensity at the filopodial tip (first three bins). Each data point represents the mean of one independent experiment and the bar graph extends to the mean of three experiments. Statistics were calculated with an unpaired two-tailed *t*-test. (F) Heatmap localization of proteins at the lamellipodia. Line scans were drawn from the cell periphery and extended approximately 1.5 µm into the cell. The fluorescence intensity of each linescan was normalized and the length binned. (G) Quantification of normalized VASP intensity at the lamellipodia (first three bins). Each data point represents the mean of one independent experiment and the bar graph extends to the mean of three experiments. Lamellipodia enrichment was calculated as the intensity at the first three bins normalized to the intensity and the subsequent fourteen bins. Statistics were calculated with a paired two-tailed *t*-test. *n*=31 (unmodified) and 33 (mmUb-K240/K286 VASP) cells across three experiments for all lamellipodia measurements.

In addition to their role at filopodia, Ena/VASP proteins also regulate actin dynamics at the lamellipodia and focal adhesions. We observed localization of both TAMRA–VASP and TAMRA–mmUb-K240/K286 to filopodial tips, lamellipodia and focal adhesions in MV^D7^ cells (Fig. 5B). To further examine this localization, we electroporated each protein into MV^D7^ cells stably expressing GFP–VASP (Fig. 6A). We observed clear colocalization of TAMRA–VASP and GFP–VASP at all three of these actin-based structures. Although TAMRA–mmUb-K240/K286 was present as well, its enrichment at each of these structures appeared to be diminished.

First, we observed a decrease in the enrichment of TAMRA–mmUb-K240/K286 at focal adhesions relative to cytoplasmic levels (Fig. 6B). Next, quantification of raw values indicated that there was significantly less mmUb-K240/K286 VASP localized to filopodia tips compared to TAMRA–VASP (Fig. 6C). To compare the distribution of unmodified VASP and mmUb-K240/K286 VASP that did localize within filopodia, we normalized VASP and actin levels along the length of filopodia. As previously reported (Applewhite et al., 2007; Rottner et al., 1999; Lebrand et al., 2004), GFP–VASP was enriched at the tips of filopodia. In contrast, phalloidin staining was most intense at the base of the filopodia, tapering in intensity towards the tip. Whereas TAMRA–VASP intensity mirrored GFP–VASP localization, the filopodial tip intensity of TAMRA–mmUb-K240/K286 was diminished (Fig. 6D,E). Similarly, linescans were utilized to evaluate VASP localization within the lamellipodia. Both GFP–VASP and TAMRA–VASP strongly colocalized at the cell periphery. Although TAMRA–mmUb-K240/K286 was still present at the lamellipodia, the fluorescence intensity was decreased relative to that of GFP–VASP. Furthermore, the ratio of fluorescence intensity at the lamellipodia to that of the adjacent medial lamella was also decreased (Fig. 6F,G). Thus, multi-monoubiquitylation of VASP reduces its protein enrichment at the filopodial tip, lamellipodia and focal adhesions.

## DISCUSSION

The actin cytoskeleton performs many essential cellular functions; thus actin polymerization must be tightly regulated. Owing to their rapid occurrence and reversible nature, post-translational modifications are ideal candidates to help modulate actin dynamics yet, with the exception of phosphorylation, are relatively understudied. Previously published work has demonstrated that non-degradative ubiquitylation of VASP reduces filopodial number and stability (Menon et al., 2015; Boyer et al., 2020). Although the mechanistic impact of VASP ubiquitylation was unclear, cellular assays suggested VASP was monoubiquitylated or multi-monoubiquitylated (Boyer et al., 2020). Here, we identified prominent ubiquitylation of VASP at K240 and K286, and generated recombinant forms of ubiquitylated VASP to interrogate actin regulatory functions. Whereas mUb-K286 did not significantly affect VASP function in any of our tested assays, monoubiquitylation of mUb-K240 significantly reduced the ability of VASP to elongate actin filaments. A multi-monoubiquitylated construct (mmUb-K240/K286) showed significant decreases in its ability to bind, bundle and elongate actin filaments, and localize to filopodia tips and lamellipodia.

### Mechanism of impairment of polymerase activity

VASP enhances actin filament elongation, reflecting its ability to bind both G-actin and F-actin through respective GAB and FAB domains (Hansen and Mullins, 2010). We observed decreases in the ability of both mUb-K240 and mmUb-K240/K286 to accelerate elongation of actin filaments. Seeded pyrene assays suggested all three ubiquitylated VASP proteins similarly associated with the barbed end of the actin filament, with a similar *k*_D_^app^ calculated for each protein. However, this interpretation is limited by the sensitivity of the pyrene assay. The 95% confidence interval for all three ubiquitylated proteins spanned a large range and we were unable to calculate an upper limit for mmUb-K240/K286. In contrast, we observed more dramatic differences in the elongation rates calculated through more sensitive single-molecule TIRFM assays. At the range of concentrations tested (1.25–25 nM), we did not observe a significantly different elongation rate for mmUb-K240/K286 compared to that for actin alone. Although this could indicate that the binding affinity of mmUb-K240/K286 for the barbed end was decreased, several lines of evidence indicate that the protein maintains a reasonable association with the barbed end. First, mmUb-K240/K286, along with both single mUb VASPs, maintained anti-capping activity, indicating interaction with the barbed end. Second, we calculated similar lifetimes for unmodified VASP and mmUb-K240/K286 bound to the barbed end. Although our measurements were limited by the speed of microscopy acquisition, we did not detect significant differences between the two proteins. Collectively, pyrene elongation assays (both in the presence and absence of capping protein) and single-molecule TIRFM assays suggest similar barbed end binding dynamics for unmodified VASP and mmUb-K240/K286.

As such, we postulate other mechanisms by which mmUb-K240/K286 could impair the acceleration of actin filament elongation. First, this deficit might arise from an impaired ability of ubiquitylated VASP to interact with monomeric actin. Of note, K240 is adjacent to the GAB and a crystal structure of this region (PDB: 2PBD) demonstrates that L234 is an essential residue for monomeric actin binding (Ferron et al., 2007). Although this structure suggests that K240 lies in an unstructured region that does not directly bind the actin monomer, ubiquitin is 8 kDa and has the potential to impair the binding between L234 and an actin monomer through steric hindrance. However, we confirmed the ability of mmUb to bind actin monomers with similar affinity to the unmodified protein through fluorescence polarization. Therefore, we hypothesize mUb-K240 might spatially impede the transfer of monomeric actin onto the filament. Interestingly, filament elongation is severely reduced with the addition of a second ubiquitylation at K286 – suggesting that the presence of additional ubiquitin moieties might further inhibit the transfer of an actin monomer onto the filament.

### Multi-monoubiquitylation of VASP regulates cell dynamics

Precise regulation of the actin cytoskeleton is required for cell morphology and motility changes. However, cells must quickly adapt to a variety of stimuli, including extracellular cues. In this work, we electroporated TAMRA–VASP and TAMRA–mmUb-K240/K286 VASP into MV^D7^ cells. In the presence of TAMRA–VASP mmUb-K240/K286, we observed changes in cell spreading morphology and intracellular localization of VASP.

Our *in vitro* results suggest that the ability of VASP to bind to the sides of actin filaments, bundle actin and enhance filament elongation is impaired by multi-monoubiquitylation. Intracellular VASP recruitment is strongly regulated by binding partners of the FPPPP motif within the EVH1 domain – such as lamellipodin, zyxin and vinculin (Krause et al., 2004; Brindle et al., 1996; Ball et al., 2000). Additional studies have suggested that the actin-binding activity of VASP might influence localization as well (Benz et al., 2009; Smolenski et al., 2000; Loureiro et al., 2002). The reduced actin side-binding activity of mmUb-VASP might account for the reduced localization of the ubiquitylated protein to actin-based structures such as the lamellipodia and focal adhesions. Furthermore, the reduced ability to enhance polymerization of actin filaments might impact the filopodial localization of mmUb-VASP and reduce the filopodial phenotype in actively spreading cells.

As VASP and TRIM9 colocalize at filopodia tips, we propose that VASP monoubiquitylation is a mechanism to rapidly control filopodial activity. However, we cannot rule out the possibility that this regulation occurs at lamellipodia and focal adhesions as well. Furthermore, this ubiquitin modification is reversible, avoiding the lengthy cycles of protein synthesis/degradation. E3 ligases exist in a balance with deubiquitylase (DUB) enzymes. Although we have demonstrated that pharmacologically blocking DUB activity prevents VASP deubiquitylation (Menon et al., 2015), the DUB for VASP is unknown. Identification of this enzyme is crucial to fully comprehend the cycle of VASP regulation in neurons, and in turn, cytoskeletal regulation.

Previously published results have demonstrated the E3 ubiquitin ligase TRIM9 is required for ubiquitylation of VASP in cultured neurons and HEK293 cells (Menon et al., 2015; Boyer et al., 2020). However, in the absence of TRIM9, residual VASP ubiquitylation remained, suggesting redundancy with other E3 ligases (Boyer et al., 2020). The discovery of additional TRIM9 substrates in developing neurons remains an open area of inquiry. Previously, we have demonstrated the netrin-1 receptor DCC is ubiquitylated in a TRIM9-dependent manner (Plooster et al., 2017). Although the type of ubiquitin modification is still unknown, molecular mass shifts and protein stability similarly suggested that it is not degradative polyubiquitylation. As DCC also localizes to the tips of filopodia (Shekarabi and Kennedy, 2002), this evokes a multi-faceted mechanism by TRIM9 to regulate filopodia function in neurons. A recent BioID experiment also identified a multitude of potential TRIM9-binding partners (Menon et al., 2021), yet additional work is required to validate these candidates as TRIM9 ubiquitylation substrates.

TRIM9 has been studied primarily in the context of neuronal development and neurodegeneration (Menon et al., 2015; Boyer et al., 2020; Plooster et al., 2017; Tanji et al., 2010; Winkle et al., 2016; Zeng et al., 2019; Do et al., 2018). However, TRIM9 is also expressed in macrophages (Tokarz et al., 2017; Carthagena et al., 2009) and various forms of cancers (Yang et al., 2020; Lin et al., 2023; Zhang et al., 2023; Mishima et al., 2015). Although the alteration of TRIM9 protein levels has been shown to affect cell proliferation, motility and inflammation in cell lines, substrates of TRIM9 have not been identified in these studies. Of note, filopodia play a crucial role in cell migration in various cancers (Jacquemet et al., 2015) and contribute to phagocytosis in macrophages (Horsthemke et al., 2017). However, whether TRIM9 localizes to filopodia – or other actin structures – in these cells and is capable of ubiquitylating VASP is unknown.

### Ubiquitylation and phosphorylation crosstalk

Post-translational modifications are essential regulators of protein stability, activity and localization. Unlike ubiquitylation, the mechanistic impact of VASP phosphorylation is well studied in numerous cell types. For example, phosphorylation of VASP at S239 and T278 by cGMP-dependent protein kinase (PKG) reduces actin polymerization *in vitro* and filamentous actin levels in HEK293 cells (Benz et al., 2009). Furthermore, work in human colon cancer carcinoma cell lines has suggested pS239 reduces VASP enrichment at filopodia tips and decreases filopodia length (Zuzga et al., 2012). Of note, crosstalk might also occur between post-translational modifications, including between ubiquitylation and phosphorylation. For example, phosphorylation of the small GTPase RhoA at Ser188 by PKA or PKG blocks ubiquitylation and proteasomal degradation of the protein (Rolli-Derkinderen et al., 2005). In contrast, phosphorylation of RhoA by the kinase Erk2 promotes RhoA ubiquitylation (Wei et al., 2013). Furthermore, our previous work suggests TRIM9-mediated ubiquitylation of DCC reduces phosphorylation at Tyr1418 (Plooster et al., 2017).

With these results in mind, we note that the VASP ubiquitylation site (K240) is located directly next to a phosphorylation site (S239) and both modifications are associated with negatively regulating VASP function. As such, we ponder this biological redundancy. However, we can speculate that that VASP mUb-K240 regulates a more specific signaling pathway compared to pS239. The kinase PKG has a prolific number of substrates and is stimulated by cGMP, a messenger with effectors throughout the body. Indeed, VASP is phosphorylated in this pathway, but it certainly will not be the only protein to be modified. In contrast, *Trim9* is predominantly expressed in neurons and glia. Furthermore, TRIM9 shows specific enrichment at the filopodial tip, providing precise local access to VASP. We have previously reported that VASP is deubiquitylated in the presence of the guidance cue netrin (Menon et al., 2015). As mentioned above, the netrin receptor DCC also localizes to the tips of filopodia (Shekarabi and Kennedy, 2002) and is ubiquitylated in TRIM9-dependent manner (Plooster et al., 2017). Collectively, TRIM9-mediated ubiquitylation might serve as a filopodial-specific negative regulator of cytoskeletal remodeling in developing neurons. However, our new work in MV^D7^ cells suggests that VASP ubiquitylation might also regulate actin dynamics at lamellipodia and focal adhesions.

### Study limitations

In this work, we chose to mimic VASP ubiquitylation with BMOE-mediated crosslinking—a sulfhydryl crosslinking approach with an 8 Å spacer arm. Although chemical ubiquitylation can be facilitated with disulfide bond (estimated at 2 Å), disulfide bonds are sensitive to reduction by DTT. As actin polymerization assays are typically performed in the presence of 0.5 M DTT to reduce the labile cysteine present in actin, we chose to use the irreversible BMOE approach. As such, we must acknowledge this increased spacer length, which may alter the impact of ubiquitylation on VASP activity.

Alternatively, we could have utilized an enzymatic approach, reconstituting the ubiquitylation reaction with purified E1, E2 and E3 ubiquitin ligases to create ubiquitylated VASP. Although our previously published work indicates that TRIM9 is likely the E3 ubiquitin ligase required for VASP ubiquitylation, we still lack much of the basic biochemical knowledge regarding TRIM9 ligase activity – including E2 partnership, post-translational modifications, and the requirement of any additional proteins. This effort to move to more physiological models of ubiquitylation is an exciting avenue of future research.

## MATERIALS AND METHODS

### Cell culture

HEK293 cells were obtained from Simon Rothenfußer (Klinikum der Universitat München, München, Germany) as previously described (Menon et al., 2015). Cells were cultured in DMEM (Gibco cat. no. 11965092) with 10% FBS (Hyclone) and maintained at 5% CO_2_ at 37°C. MV^D7^ cells (Bear et al., 2000) were a gift from Frank Gertler (MIT, MA, USA). Cells were maintained in DMEM supplemented with GlutaMAX (Thermo Fisher Scientific, cat. no. 35050061), 15% FBS (Thermo Fisher cat. no. FB12999102), and 50 U/mL of interferon-γ (Sigma cat. no. IF005). Cells were cultured at 32°C with 5% CO_2_. Cells lines are routinely tested for Mycoplasma by qPCR.

### Plasmid transfections

HEK293 cells were transfected with Lipofectamine 2000 (Thermo Fisher Scientific) according to the manufacturer's instructions.

### Plasmids, antibodies, and reagents

Creation of Myc-VASP was described previously (Menon et al., 2015; Boyer et al., 2020). pRK5-HA-Ubiquitin-WT was Addgene plasmid #17608 (deposited by Ted Dawson; Lim et al., 2005). pGEXTEV Flag PS-SSSS-UB^G75C^ creation was previously described (Brown et al., 2016).

Cysteine-light His–VASP (His–VASP^CCC-SSA^) containing an N-terminal KCK motif was a gift from Dyche Mullins (UCSF, CA, USA; Hansen and Mullins, 2010). To facilitate sortase labeling, the KCK motif was mutated to a GGG motif using QuikChange mutagenesis. VASP K240C and VASP K286C were also generated using QuikChange mutagenesis. VASP K240C, K286C was created by Azenta Life Sciences through site-directed mutagenesis.

Primary antibodies used were: mouse monoclonal against c-Myc (9E10) [1:2000 for western blotting (WB), Hybridoma serum purified in-house], rabbit polyclonal against the HA tag (1:250 WB, Thermo Fisher Scientific cat. no. 71-5500), rabbit polyclonal against VASP (Sigma-Aldrich, cat. no. V3390, 1:1000 WB), and mouse monoclonal against β-actin (Proteintech cat. no. 66009, 1:10,000 WB). Fluorescent secondary antibodies included IRDye 680LT goat anti-rabbit IgG secondary antibody (LI-COR cat. no. NC9030093, 1:10,000 WB) and IRDye 800CW goat anti-mouse IgG secondary antibody (LI-COR cat. no. NC9401841, 1:10,000 WB).

Other reagents included PR-619 (Thermo Fisher Scientific cat. no. 66-214-125), MG132 (81-5-15, American Peptide Company), Alexa Fluor™ 488 phalloidin (Thermo Fisher Scientific cat. no. A12379), Alexa Fluor™ 647 phalloidin (Thermo Fisher Scientific cat. no. A22287), bismaleimidoethane (Thermo Fisher Scientific cat. no. 22323) and paraformaldehyde (Thermo Fisher Scientific cat. no. PI28908).

### Ubiquitylation assay and immunoblotting

The ubiquitylation assay was performed as described previously (Menon et al., 2015; Boyer et al., 2020). Cells were treated with MG-132 (81-5-15, American Peptide Company) for 1 h before lysis in buffer containing 20 mM Tris-HCl, 250 mM NaCl, 3 mM EDTA, 3 mM EGTA, 0.5% NP-40, 1% SDS, 2 mM DTT, 5 mM *N*-ethylmaleimide, protease and phosphatase inhibitors. Lysates were vortexed, boiled for 20 min, cleared at 20,000 ***g*** for 10 min and diluted in lysis buffer lacking SDS to bring the final SDS concentration to 0.1%. Anti-Myc monoclonal antibody was added and incubated overnight at 4°C with rocking. Protein A beads were added in the morning and incubated for 2 h. After washing twice with lysis buffer, beads were resuspended in 1× Laemmli sample buffer. Samples were loaded on a 7.5% SDS-PAGE gel, transferred on nitrocellulose membrane for 90 min (75 V), blocked in 5% milk in TBS for 1 h (at room temperature), and incubated with primary antibody overnight in 1% milk in Tris-buffered saline with 0.1% Tween-20 (TBST). Membranes were washed three times (5 min) in TBST, incubated with secondary antibodies for 1 h, washed 3× (10 min) in TBST, rinsed in PBS and imaged on an Odyssey (LI-COR Biosciences). Uncropped images of western blots are presented in Fig. S5.

### Mass spectrometry

Two biological replicates – each containing four technical replicates – were prepared for proteomic analysis of Myc–VASP ubiquitylation sites. HEK293 cells (700,000 total) were seeded on a 10 cm diameter tissue culture dish and transfected the next day with 1.5 µg of Myc–VASP using Lipofectamine 2000 (Invitrogen cat. no. 11668027) according to the manufacturer's instructions. The day after transfection, cells were treated with 10 µM of MG-132 for 1 h. Cells were rotated in lysis buffer (10 min, 4°C) and cleared at 20,000 ***g*** (10 min, 4°C). Lysis buffer was composed of 50 mM Tris-HCl pH 7.4, 150 mM NaCl, 1 mM EDTA, 0.5% Triton X-100, 150 µg/ml PMSF, 2 µg/ml leupeptin, 5 µg/ml aprotinin, 15 mM sodium pyrophosphate, 50 mM NaF, 40 mM β-gly, 1 mM sodium vanadate, 10 mM N-ethylmaleimide (NEM) and 50 µM PR-619.

Lysate was incubated with equilibrated Myc-Trap agarose beads (Chromotek, Planegg, Germany, cat. no. yta) for 2 h at 4°C to enrich for Myc–VASP. Beads were spun down at 200 ***g***, washed once with lysis buffer, and washed three times with wash buffer (lysis buffer containing 500 mM NaCl and 1% Triton X-100) at 4°C. After a final brief wash with 1% SDS in PBS at room temperature, beads were resuspended in 1× sample buffer (Bio-Rad) with 50 mM DTT and boiled for 10 min at 98°C.

Immunoprecipitated samples were subjected to SDS-PAGE and stained with Colloidal Coomassie (Invitrogen). The bands corresponding to VASP were excised and the proteins were reduced with 10 mM DTT for 30 min at room temperature, alkylated with 100 mM iodoacetamide for 45 min in the dark at room temperature, and in-gel digested with either ArgC (Promega) overnight at 37°C or trypsin (Promega) for 1 h at 37°C. Peptides were extracted, desalted with C18 spin columns (Pierce) and dried via vacuum centrifugation. Peptide samples were stored at −80°C until further analysis.

The peptide samples (*n*=2) were analyzed in technical duplicate by LC-MS/MS using an Easy nLC 1200 coupled to a QExactive HF mass spectrometer (Thermo Fisher Scientific). Samples were injected onto an Easy Spray PepMap C18 column (75 μm id×25 cm, 2 μm particle size) (Thermo Fisher Scientific) and separated over a 45 min method. The gradient for separation consisted of 5–38% mobile phase B at a 250 nl/min flow rate, where mobile phase A was 0.1% formic acid in water and mobile phase B consisted of 0.1% formic acid in 80% acetonitrile (ACN). The QExactive HF was operated in data-dependent mode where the 15 most intense precursors were selected for subsequent fragmentation. Resolution for the precursor scan (*m*/*z* 350–1600) was set to 60,000 with a target value of 3×10^6^ ions. MS/MS scan resolution was set to 15,000 with a target value of 1×10^5^ ions, 100 ms injection time. The normalized collision energy was set to 27% for HCD. Dynamic exclusion was set to 30 s, peptide match was set to preferred, and precursors with unknown charge or a charge state of 1 and ≥8 were excluded.

Raw data files were processed using Proteome Discoverer version 2.5 (Thermo Fisher Scientific). Peak lists were searched against the reviewed UniProt human database, appended with a common contaminants database, using Sequest. The following parameters were used to identify tryptic or ArgC peptides: 50 ppm precursor ion mass tolerance; 0.02 Da product ion mass tolerance; up to three missed trypsin cleavage sites; (C) carbamidomethylation was set as a fixed modification; (M) oxidation, (S, T, Y) phosphorylation, and (K) diGly were set as variable modifications. The ptmRS node was used to localize the sites of phosphorylation and diGly sites. Peptide false discovery rates (FDRs) were calculated by the Percolator node using a decoy database search and peptides were filtered using a 1% FDR cutoff. MS/MS spectrum was annotated using IPSA (van der Wal et al., 2018). MS Data are available via ProteomeXchange (Perez-Riverol et al., 2022, 2016; Deutsch et al., 2023) with identifier PXD041196.

### Protein purification

GST–Ubiquitin^1-74,G75C^ was transformed into BL21-CodonPlus (DE3)-RIL competent cells (Agilent Technologies cat. no. 230245) and grown in LB medium with 200 µg/ml of ampicillin and 25 µg/ml of chloramphenicol. Cultures were grown at 37°C until the optical density at 600 nm (OD) reached 0.8–1. IPTG (0.6 mM) was added and cultures were grown overnight at 18°C. Bacteria were pelleted (4000 ***g*** for 10 min), resuspended in lysis buffer (50 mM Tris-HCl pH 8, 200 mM NaCl, 1 mM DTT and 2.5 mM PMSF), and frozen until use. Pellets were thawed, lysed by Emulsiflex and centrifuged for 40 min (36,600 ***g***, SS-34 rotor). Protein was bound to Pierce glutathione agarose (Thermo Fisher Scientific, 16101) and washed twice with four column volumes (CVs) of wash buffer (10 mM Tris-HCl, pH 8, 250 mM NaCl and 1 mM DTT). Hrv-3c protease (purified in house by affinity chromatography from pGEX4T-1 PreCission) was added at a 1:200 ratio in wash buffer to perform on-bead cleavage of the protein.

His–VASP was transformed into BL21-CodonPlus (DE3)-RIL competent cells and grown in LB medium with 200 µg/ml of ampicillin and 25 µg/ml of chloramphenicol. Cultures were grown at 37°C until the OD reached 0.8–1. IPTG (0.6 mM) was added and cultures were grown overnight at 18°C. Bacteria were pelleted (4000 ***g*** for 10 min), resuspended in lysis buffer (50 mM HEPES pH 7.4, 300 mM NaCl, 10 mM imidazole, 10 mM β-mercaptoethanol) and frozen until use. Pellets were thawed, lysed by sonication and cleared with a high-speed spin (36,600 ***g*** for 40 min). His–VASP was bound to HisPur™ Ni-NTA Resin (Thermo Fisher Scientific 88221), washed twice with 5 CVs of wash buffer (50 mM HEPES pH 7.4, 300 mM NaCl, 30 mM imidazole, 10 mM β-mercaptoethanol), and eluted with 4 CVs of 50 mM HEPES pH 7.4, 300 mM NaCl, 250 mM imidazole, 10 mM β-mercaptoethanol. Eluted His–VASP was dialyzed overnight in 20 mM HEPES pH 7.4, 200 mM NaCl, and 1 mM DTT before separation over a Superose 6 column (GE) on an AKTA FPLC (GE Healthcare).

Actin was purified from chicken skeletal-muscle acetone powder as described previously (Spudich et al., 1971). A portion of actin was then labeled on lysine residues with Alexa Fluor 488-succinimidylester as described previously (Isambert et al., 1995).

Human fascin and mouse capping protein (dual-expression construct of MmCP α1-His(6×)-tag and SNAP-β2; SNAP-CP) were expressed in bacteria and purified as previously described (Vignjevic et al., 2006; Burke et al., 2017).

Murine profilin-1 (pMW-MmPRF-1) was transformed into BL21-CodonPlus(De3)-RP competent cells (Agilent Technologies cat. no. 230255). Cultures were grown at 37°C until the OD reached 0.6–0.8, IPTG (0.5 mM) was added, and cultures were grown for 5 h at 37°C. Bacteria were pelleted (4500 ***g*** for 10 min), resuspended in lysis buffer (50 mM Tris-HCl pH 8, 200 mM NaCl, 1 mM DTT and 2.5 mM PMSF) and frozen until use. Pellets were thawed, lysed in 20 mM Tris-HCl, pH 7.5, 150 mM KCl and 0.2 mM DTT, and cleared. Profilin was bound to a poly-proline column, washed with 30 mL of lysis buffer, washed with 30 ml of 2 M urea, and eluted with 7 M urea. Purified profilin was dialyzed overnight in lysis buffer, centrifuged to pellet any precipitated protein (15,200 ***g*** for 30 min), concentrated, and flash frozen until use.

### Crosslinking

Crosslinking protocols were adapted from previously published work (Brown et al., 2015). His–VASP constructs and ubiquitin G75C were reduced with 10 mM DTT and buffer exchanged into reaction buffer (50 mM HEPES pH 7.0, 200 mM NaCl). Ubiquitin was modified with 10-molar excess BMOE (Thermo Fisher Scientific cat. no. 22323) for 30 min on ice and desalted into fresh reaction buffer. A 10-fold excess of ubiquitin-BMOE was added to His–VASP K240C and His–VASP K286C, with a 20-fold excess added to His–VASP K240C, K286C. After a 1-h incubation (on ice), the reaction was quenched with 10 mM β-mercaptoethanol.

To remove excess (non-crosslinked) ubiquitin, His–VASP was re-purified by nickel affinity chromatography. Protein was buffer exchanged to remove excess imidazole and incubated with His-TEV overnight at 4°C.

### Sortase labeling

A custom peptide (TAMRA–PEG6–LPETGG) was purchased from BioMatik (Wilmington, Delaware) to facilitate fluorescent labeling of VASP. After confirming cleavage of the His tag from VASP using TEV protease, the sortase reaction was set up with a 20-fold excess of sortase peptide and 2.5 µM His-sortase (purified in house by affinity chromatography) in a reaction buffer (50 mM HEPES, pH 7.4, 200 mM NaCl, 10 mM CaCl_2_). After 1 h incubation (on ice), the reaction was quenched with 20 mM EGTA and buffer-exchanged to remove excess peptide. His-TEV and His-sortase were removed following incubation with nickel resin. TAMRA–VASP ubiquitylation mimics were further purified over a Superose 6 column. Purified protein was stored in 10 mM HEPES, 200 mM NaCl, 1 mM DTT and 10% glycerol, aliquoted and flash frozen.

### Mass photometry

Measurements were completed on a Refeyn One Mass Photometer (Refeyn Ltd, Oxford, UK) and analyzed using DiscoverMP software (Refeyn Ltd) as previously described (Cannon et al., 2023). Briefly, imaging wells were assembled from imaging coverslips (24×50 mm^2^, Thorlabs) and adhesive four-well gaskets (Thorlabs). Measurements were acquired for 1 min at a frame rate of 100 fps at room temperature using AcquireMP software (Refeyn Ltd) using 15 nM VASP. We calculated ∼20–25% margin of error for each estimated molecular mass.

### Actin co-sedimentation assays

Co-sedimentation assays were adapted from previously described protocols (Zimmermann et al., 2016). Lyophilized rabbit skeletal muscle actin (99% pure, Cytoskeleton Inc., Denver, Colorado, cat. no. AKL99) was resuspended in water and diluted to 10 mg/ml in fresh Ca-G buffer (2 mM Tris-HCl pH 8, 0.2 mM ATP, 0.1 mM CaCl_2_, 0.5 mM DTT and 0.01% NaN_3_). After a 1 h incubation on ice to depolymerize actin oligomers, actin was centrifuged (20,000 ***g***, 4°C) for 20 min, and the concentration was confirmed. Actin polymerization was induced by the addition of 10 mM HEPES pH 7.4, 10 mM EGTA, 10 mM MgCl_2_, 50 mM NaCl and 1 mM DTT and filaments were incubated for 1 h at room temperature.

For low-speed sedimentation assays, varying amounts of VASP (100–600 nM) and actin filaments (2 µM) then incubated for 1 h at room temperature before centrifugation at 10,000 ***g*** (room temperature) in a tabletop centrifuge. The top 50% of the supernatant was removed, mixed with Laemmli sample buffer and boiled. The remaining 50% of the supernatant was discarded. The pellet was washed carefully in 10 mM HEPES (pH 7.4), 10 mM EGTA, 10 mM MgCl_2_, 50 mM NaCl, and 1 mM DTT. The pellet was then directly resuspended in Laemmli sample buffer, transferred to an Eppendorf tube and boiled. For high-speed sedimentation assays, various amounts of VASP (200–600 nM) and actin filaments (1 µM) then incubated for 1 h at room temperature before centrifugation at 100,000 ***g*** (23°C) in a TLA100 rotor (Beckman-Coulter). The top 50% of the supernatant was removed, mixed with 4× SB, and boiled. The remaining 50% of the supernatant was discarded. The pellet was washed carefully in 10 mM HEPES pH 7.4, 10 mM EGTA, 10 mM MgCl_2_, 50 mM NaCl and 1 mM DTT. The pellet was then directly resuspended in 1× SB, transferred to an Eppendorf tube and boiled. Supernatant and pellet fractions were separated on SDS-PAGE gels and stained with Colloidal Coomassie (Invitrogen) for 3 h. After destaining in water, gels were scanned on a Licor Odyssey and analyzed by densitometry in ImageStudioLite.

### Pyrene actin elongation assays

Pyrene elongation assays were adapted from previously described protocols (Zimmermann et al., 2016). Before starting, both rabbit skeletal muscle actin (Cytoskeleton cat. no. AKL99) and pyrene-labeled rabbit skeletal muscle actin (Cytoskeleton cat. no. AP05) were spun down at 100,000 ***g*** for 1 h at 4°C in a TLA100 rotor (Beckman-Coulter, Indianapolis, Indiana). After conversion of Ca^2+^-ATP-actin into Mg^2+^-ATP-actin, polymerization of actin filaments (5 µM) was initiated by adding in 10 mM HEPES pH 7.4, 10 mM EGTA, 10 mM MgCl_2_, 50 mM NaCl and 1 mM DTT. Filaments polymerized for 1 h at 23°C. Additional proteins (VASP and/or capping protein) were added to a final concentration of 0.5 µM filaments before the addition of 0.5 µM actin monomers (20% pyrene labeled). Pyrene fluorescence was measured with a Spark Cyto (Tecan, Männedorf, Switzerland) or a CLARIOstar (BMG Labtech, Ortenberg, Germany) plate reader.

Pyrene assays with profilin were completed with a final concentration of 1 µM filaments and 0.5 µM monomers in the presence of 5 µM profilin and 40 nM VASP. Pyrene assays examining elongation in the presence of both capping protein and profilin were completed with 1 µM actin filaments, 0.5 µM actin monomers, 1 µM profilin, 5 nM capping protein and 200 nM VASP.

### Microscope descriptions

TIRFM images were acquired using an Olympus IX-71 microscope through TIRF illumination, recorded with a iXon EMCCD camera (Andor Technology) and a cellTIRF-4Line system (Olympus).

All widefield imaging was performed using an inverted microscope (IX83-ZDC2; Evident/Olympus) equipped with a cMOS camera (Orca-Fusion, Hamamatsu) and xenon light source. Images were acquired using a 100×1.50 NA TIRF objective (Olympus) and with Cellsens software (Evident/Olympus).

Confocal microscopy was performed on an inverted laser scanning confocal microscopy (LSM980, Zeiss) with a motorized *x,y* stage and *z* focus with high speed Piezo insert. The microscope is equipped with four diode lasers (405, 488, 561 and 633 nm). Images were acquired with 1× Nyquist sampling using a 63×1.4 NA Plan-Apochromat oil objective (Zeiss) and four-channel GaAsP detectors on Zen Blue 3.6 software. This microscope is equipped with Airyscan 2; however, this module was not used in these experiments.

### TIRFM assays

Microscope slides and coverslips (#1.5; Thermo Fisher Scientific, Waltham, MA) were sonicated for 2 h with Helmanex III detergent (Hellma Analytics, Müllheim, Germany). After rinsing in deionized water and drying, glass was incubated with 1 mg/ml mPeg-Silane (5000 MW; PLS-201, Creative PEGWorks) in 95% ethanol, pH 2.0 overnight while shaking. Imaging flow chambers were created using double-sided tape to form parallel wells (Zimmermann et al., 2016).

All TIRF assays utilized purified chicken muscle actin. Spontaneous actin assembly was initiated by mixing 1.5 µM Mg-actin monomers (10% AF488 labeled) with 1× TIRF buffer [10 mM imidazole pH 7.0, 50 mM KCl, 1 mM MgCl_2_, 1 mM EGTA, 50 mM DTT, 0.2 mM ATP, 50 μM CaCl_2_, 15 mM glucose, 20 μg/ml catalase, 100 μg/ml glucose oxidase and 0.5% (400 cP) methylcellulose], as well as any additional proteins such as VASP and fascin. Samples were then transferred to flow chambers and imaged at room temperature by TIRFM.

Actin elongation rates were determined by manually tracking the elongation of single actin filaments or parallel actin bundles in FIJI and the average elongation rate for each filament was then calculated. Researchers were aware of the experimental conditions for the determinations.

Quantification of 1 nM VASP at the barbed ends of actin filaments (in the presence of 673 nM fascin) was adapted from previously published work (Harker et al., 2019). VASP residence was specifically quantified on the trailing filaments of two-filament bundles. Linescans were used to trace actin filaments and kymographs were created in ImageJ (Schindelin et al., 2012) to visualize the growth of the trailing barbed end. Dynamic VASP puncta binding was quantified; stationary puncta were excluded, as they were assumed to be adhered to the coverslip. Likewise, puncta on actin bundles where the trailing versus leading filament was not evident were not included. Cumulative frequency of dwell times were calculated and (1−cumulative frequency) was fit with a one-phase decay curve as described previously (Hansen and Mullins, 2010).

Analysis of actin filament bundles was completed in ImageJ (Schindelin et al., 2012). The ridge detection plugin was used to trace actin filaments and bundles at three timepoints (0, 5, and 10 min) in each TIRF movie. Detected filaments shorter than 0.5 µm were excluded from analysis. The mean grey value of AF488–actin was measured for each filament/bundle. This mean fluorescence intensity of each bundle was normalized to the mean intensity of a single actin filament to estimate the number of actin filaments per bundle.

### Fluorescence polarization

AF488-labeled actin monomers were diluted in fresh G-buffer (2 mM Tris-HCl pH 8, 0.2 mM ATP, 0.1 mM CaCl_2_, 0.5 mM DTT and 0.01% NaN_3_) supplemented with 50 µM MgCl_2_ and 0.2 mM EGTA. VASP (5% volume of the final reaction) was mixed with the actin. Reactions were incubated in the dark for 30 min at room temperature before the 96-well plate was read. VASP buffer consisted of 40 mM HEPES, 180 mM NaCl, 0.9 mM DTT plus 10% glycerol. The final salt concentration of reaction is estimated at 9 mM NaCl.

Fluorescence polarization was measured with a CLARIOstar (BMG Labtech) plate reader. Settings include: excitation (486-16), dichroic filter (LP 504), emission (530-40). The target mP value was set at 35 mP.

### Neon electroporation of MV^D7^ cells

25 mm #1.5 German glass coverslips (Electron Microscopy Services cat. no. 72290-12) ­were cleaned with a Harrick Plasma Cleaner (PDC-32G). Coverslips were coated with 10 µg/ml fibronectin (Corning cat. no. 356008), incubated for 1 h at 32°C, and washed before cells were plated.

MV^D7^ were electroporated using the Neon™ Transfection System (Thermo Fisher Scientific MPK5000). Briefly, cells were grown to 70–90% confluency, removed from the dish with accutase (Sigma-Aldrich cat. no. A6964), washed with PBS, and resuspended in Neon™ Resuspension Buffer R at a final density of 0.5×10^8^ cells/ml. In the final 10 µl Neon reaction, 1.1 µM of protein and 440,000 cells were used. Neon settings were pulse voltage (1250), pulse width (30 ms), pulse number (1).

220,000 cells were plated per coverslip and incubated for 30 min at 32°C. Cells were fixed by adding equal volumes of warm 8% PFA in 2× PHEM buffer (120 mM PIPES, 50 mM HEPES, 20 mM EGTA, 4 mM MgSO_4_ and 0.24 M sucrose) directly to the medium for 20 min at room temperature. Coverslips were washed three times with PBS.

After fixation, cells were permeabilized with 0.1% Triton X-100 for 10 min and blocked with 10% donkey serum (Thermo Fisher Scientific cat. no. OB003001) for 30 min. To visualize actin filaments, cells were incubated with Alexa Fluor 488- or 647-conjugated phalloidin at 1:400 or 1:200 (respectively) for 1 h at room temperature. Coverslips were washed three with PBS, mounted in a Tris, glycerol and *n*-propyl-gallate-based mounting medium made in house and sealed with nail polish.

### MV^D7^ cell analysis

All images were processed and analyzed in ImageJ (Schindelin et al., 2012).

For classification of widefield cell spreading images, conditions were hidden from the researcher by using the File Name Encrypter plugin before analysis. Cell masks were created using the phalloidin channel to quantify cell area, perimeter and fluorescence intensity. Cell classification was based on the presence of a smooth and even lamellipodia (smooth), a filopodial density greater than 0.1 filopodia/µm perimeter (filopodial) and the presence of actin ruffles (ruffled), as previously described (Applewhite et al., 2007).

Maximum projections were created from confocal images to analyze lamellipodia, filopodia and focal adhesion protein enrichment. Images were manually background subtracted using an average of blank regions on the coverslip. GFP–VASP images were used to draw linescans on each structure. To account for variability in lamellipodia thickness, four linescans were drawn per cell in areas with clear lamellipodia (per GFP–VASP localization). Filopodia linescans were drawn from the filopodia tip to the base. Raw filopodial tip intensity values were calculated by averaging the first four pixels (0.255 µm) of the filopodia. Heatmaps displaying protein localization were created based on FiloMap code (Jacquemet, 2023). For focal adhesions, masks of the structures were created based on the GFP–VASP channel. The mean fluorescence intensity was measured and normalized to the mean cytoplasmic fluorescence intensity of the cell (measured by averaging three different 2×2 µm regions within the cell).

### Software

Microscopy images were visualized and analyzed in ImageJ (Schindelin et al., 2012). Western blot images were visualized and analyzed in ImageStudioLite (LI-COR Biosciences). Data visualization and statistical analysis were completed in Prism 9 (GraphPad). Figures were created in Adobe Illustrator.

## Supplementary Material



10.1242/joces.261527_sup1 Supplementary information

## References

[JCS261527C1] Applewhite, D. A., Barzik, M., Kojima, S.-I., Svitkina, T. M., Gertler, F. B. and Borisy, G. G. (2007). Ena/VASP proteins have an anti-capping independent function in Filopodia formation. *Mol. Biol. Cell* 18, 2579-2591. 10.1091/mbc.e06-11-0990 17475772 PMC1924831

[JCS261527C2] Baker, R., Wilkerson, E. M., Sumita, K., Isom, D. G., Sasaki, A. T., Dohlman, H. G. and Campbell, S. L. (2013). Differences in the regulation of K-Ras and H-Ras isoforms by monoubiquitination. *J. Biol. Chem.* 288, 36856-36862. 10.1074/jbc.C113.525691 24247240 PMC3873545

[JCS261527C3] Ball, L. J., Kühne, R., Hoffmann, B., Häfner, A., Schmieder, P., Volkmer-Engert, R., Hof, M., Wahl, M., Schneider-Mergener, J. and Walter, U. (2000). Dual epitope recognition by the VASP EVH1 domain modulates polyproline ligand specificity and binding affinity. *EMBO J.* 19, 4903-4914. 10.1093/emboj/19.18.4903 10990454 PMC314220

[JCS261527C4] Barzik, M., Kotova, T. I., Higgs, H. N., Hazelwood, L., Hanein, D., Gertler, F. B. and Schafer, D. A. (2005). Ena/VASP proteins enhance actin polymerization in the presence of barbed end capping proteins. *J. Biol. Chem.* 280, 28653-28662. 10.1074/jbc.M503957200 15939738 PMC1747414

[JCS261527C5] Bear, J. E. and Gertler, F. B. (2009). Ena/VASP: towards resolving a pointed controversy at the barbed end. *J. Cell Sci.* 122, 1947-1953. 10.1242/jcs.038125 19494122 PMC2723151

[JCS261527C6] Bear, J. E., Loureiro, J. J., Libova, I., Fässler, R., Wehland, J. and Gertler, F. B. (2000). Negative regulation of fibroblast motility by Ena/VASP proteins. *Cell* 101, 717-728. 10.1016/S0092-8674(00)80884-3 10892743

[JCS261527C7] Bear, J. E., Svitkina, T. M., Krause, M., Schafer, D. A., Loureiro, J. J., Strasser, G. A., Maly, I. V., Chaga, O. Y., Cooper, J. A., Borisy, G. G. et al. (2002). Antagonism between Ena/VASP proteins and actin filament capping regulates fibroblast motility. *Cell* 109, 509-521. 10.1016/S0092-8674(02)00731-6 12086607

[JCS261527C8] Benz, P. M., Blume, C., Seifert, S., Wilhelm, S., Waschke, J., Schuh, K., Gertler, F., Munzel, T. and Renne, T. (2009). Differential VASP phosphorylation controls remodeling of the actin cytoskeleton. *J. Cell Sci.* 122, 3954-3965. 10.1242/jcs.044537 19825941 PMC2773194

[JCS261527C9] Boyer, N. P., McCormick, L. E., Menon, S., Urbina, F. L. and Gupton, S. L. (2020). A pair of E3 ubiquitin ligases compete to regulate filopodial dynamics and axon guidance. *J. Cell Biol.* 219, e201902088. 10.1083/jcb.201902088 31820781 PMC7039193

[JCS261527C10] Breitsprecher, D., Kiesewetter, A. K., Linkner, J., Urbanke, C., Resch, G. P., Small, J. V. and Faix, J. (2008). Clustering of VASP actively drives processive, WH2 domain-mediated actin filament elongation. *EMBO J.* 27, 2943-2954. 10.1038/emboj.2008.211 18923426 PMC2585163

[JCS261527C11] Breitsprecher, D., Kiesewetter, A. K., Linkner, J., Vinzenz, M., Stradal, T. E. B., Small, J. V., Curth, U., Dickinson, R. B. and Faix, J. (2011). Molecular mechanism of Ena/VASP-mediated actin-filament elongation. *EMBO J.* 30, 456-467. 10.1038/emboj.2010.348 21217643 PMC3034019

[JCS261527C12] Brindle, N. P., Holt, M. R., Davies, J. E., Price, C. J. and Critchley, D. R. (1996). The focal-adhesion vasodilator-stimulated phosphoprotein (VASP) binds to the proline-rich domain in vinculin. *Biochem. J.* 318, 753-757. 10.1042/bj3180753 8836115 PMC1217682

[JCS261527C13] Brown, N. G., VanderLinden, R., Watson, E. R., Qiao, R., Grace, C. R. R., Yamaguchi, M., Weissmann, F., Frye, J. J., Dube, P., Ei Cho, S. et al. (2015). RING E3 mechanism for ubiquitin ligation to a disordered substrate visualized for human anaphase-promoting complex. *Proc. Natl. Acad. Sci. USA* 112, 5272-5279. 10.1073/pnas.1504161112 25825779 PMC4418899

[JCS261527C14] Brown, N. G., VanderLinden, R., Watson, E. R., Weissmann, F., Ordureau, A., Wu, K. P., Zhang, W., Yu, S., Mercredi, P. Y., Harrison, J. S. et al. (2016). Dual RING E3 architectures regulate multiubiquitination and ubiquitin chain elongation by APC/C. *Cell* 165, 1440-1453. 10.1016/j.cell.2016.05.037 27259151 PMC4991212

[JCS261527C15] Brühmann, S., Ushakov, D. S., Winterhoff, M., Dickinson, R. B., Curth, U. and Faix, J. (2017). Distinct VASP tetramers synergize in the processive elongation of individual actin filaments from clustered arrays. *Proc. Natl. Acad. Sci. USA* 114, E5815-E5824. 10.1073/pnas.1703145114 28667124 PMC5530675

[JCS261527C69] Burke, T. A., Harker, A. J., Dominguez, R. and Kovar, D. R. (2017). The bacterial virulence factors VopL and VopF nucleate actin from the pointed end. *J. Cell Biol.* 216, 1267-1276. 10.1083/jcb.201608104 28363971 PMC5412564

[JCS261527C16] Cannon, K. S., Sarsam, R. D., Tedamrongwanish, T., Zhang, K. and Baker, R. W. (2023). Lipid nanodiscs as a template for high-resolution cryo-EM structures of peripheral membrane proteins. *J. Struct. Biol.* 215, 107989. 10.1016/j.jsb.2023.107989 37364761

[JCS261527C17] Carthagena, L., Bergamaschi, A., Luna, J. M., David, A., Uchil, P. D., Margottin-Goguet, F., Mothes, W., Hazan, U., Transy, C., Pancino, G. et al. (2009). Human TRIM gene expression in response to interferons. *PLoS One* 4, e4894. 10.1371/journal.pone.0004894 19290053 PMC2654144

[JCS261527C18] Cooper, J. A. and Pollard, T. D. (1985). Effect of capping protein on the kinetics of actin polymerization. *Biochemistry* 24, 793-799. 10.1021/bi00324a039 3994986

[JCS261527C19] Dent, E. W., Kwiatkowski, A. V., Mebane, L. M., Philippar, U., Barzik, M., Rubinson, D. A., Gupton, S., Van Veen, J. E., Furman, C., Zhang, J. et al. (2007). Filopodia are required for cortical neurite initiation. *Nat. Cell Biol.* 9, 1347-1359. 10.1038/ncb1654 18026093

[JCS261527C20] Deutsch, E. W., Bandeira, N., Perez-Riverol, Y., Sharma, V., Carver, J. J., Mendoza, L., Kundu, D. J., Wang, S., Bandla, C., Kamatchinathan, S. et al. (2023). The ProteomeXchange consortium at 10 years: 2023 update. *Nucleic Acids Res.* 51, D1539-D1548. 10.1093/nar/gkac1040 36370099 PMC9825490

[JCS261527C21] Do, L. D., Gupton, S. L., Tanji, K., Bastien, J., Brugière, S., Couté, Y., Quadrio, I., Rogemond, V., Fabien, N., Desestret, V. et al. (2018). TRIM9 and TRIM67 are new targets in paraneoplastic cerebellar degeneration. *Cerebellum* 18, 245-254. 10.1007/s12311-018-0987-5 PMC6445697 30350014

[JCS261527C22] Faix, J. and Rottner, K. (2022). Ena/VASP proteins in cell edge protrusion, migration and adhesion. *J. Cell Sci.* 135, jcs259226. 10.1242/jcs.259226 35285496

[JCS261527C23] Ferron, F., Rebowski, G., Lee, S. H. and Dominguez, R. (2007). Structural basis for the recruitment of profilin-actin complexes during filament elongation by Ena/VASP. *EMBO J.* 26, 4597-4606. 10.1038/sj.emboj.7601874 17914456 PMC2063483

[JCS261527C24] Gupton, S. L. and Gertler, F. B. (2010). Integrin signaling switches the cytoskeletal and exocytic machinery that drives neuritogenesis. *Dev. Cell* 18, 725-736. 10.1016/j.devcel.2010.02.017 20493807 PMC3383070

[JCS261527C25] Hansen, S. D. and Mullins, R. D. (2010). VASP is a processive actin polymerase that requires monomeric actin for barbed end association. *J. Cell Biol.* 191, 571-584. 10.1083/jcb.201003014 21041447 PMC3003327

[JCS261527C26] Harker, A. J., Katkar, H. H., Bidone, T. C., Aydin, F., Voth, G. A., Applewhite, D. A. and Kovar, D. R. (2019). Ena/VASP processive elongation is modulated by avidity on actin filaments bundled by the filopodia cross-linker fascin. *Mol. Biol. Cell* 30, 851-862. 10.1091/mbc.E18-08-0500 30601697 PMC6589784

[JCS261527C27] Horsthemke, M., Bachg, A. C., Groll, K., Moyzio, S., Müther, B., Hemkemeyer, S. A., Wedlich-Söldner, R., Sixt, M., Tacke, S., Bähler, M. et al. (2017). Multiple roles of filopodial dynamics in particle capture and phagocytosis and phenotypes of Cdc42 and Myo10 deletion. *J. Biol. Chem.* 292, 7258-7273. 10.1074/jbc.M116.766923 28289096 PMC5409491

[JCS261527C70] Isambert, H., Venier, P., Maggs, A. C., Fattoum, A., Kassab, R., Pantaloni, D. and Carlier, M. F. (1995). Flexibility of actin filaments derived from thermal fluctuations. Effect of bound nucleotide, phalloidin, and muscle regulatory proteins. *J. Biol. Chem.* 270, 11437-11444. 10.1074/jbc.270.19.11437 7744781

[JCS261527C28] Jacquemet, G. (2023). Mapping the localization of proteins within Filopodia using FiloMap. *Methods Mol. Biol.* 2608, 51-61. 10.1007/978-1-0716-2887-4_4 36653701

[JCS261527C29] Jacquemet, G., Hamidi, H. and Ivaska, J. (2015). Filopodia in cell adhesion, 3D migration and cancer cell invasion. *Curr. Opin. Cell Biol.* 36, 23-31. 10.1016/j.ceb.2015.06.007 26186729

[JCS261527C30] Krause, M., Dent, E. W., Bear, J. E., Loureiro, J. J. and Gertler, F. B. (2003). Ena/VASP proteins: regulators of the actin cytoskeleton and cell migration. *Annu. Rev. Cell Dev. Biol.* 19, 541-564. 10.1146/annurev.cellbio.19.050103.103356 14570581

[JCS261527C31] Krause, M., Leslie, J. D., Stewart, M., Lafuente, E. M., Valderrama, F., Jagannathan, R., Strasser, G. A., Rubinson, D. A., Liu, H., Way, M. et al. (2004). Lamellipodin, an Ena/VASP ligand, is implicated in the regulation of lamellipodial dynamics. *Dev. Cell* 7, 571-583. 10.1016/j.devcel.2004.07.024 15469845

[JCS261527C32] Kwiatkowski, A. V., Rubinson, D. A., Dent, E. W., Edward van Veen, J., Leslie, J. D., Zhang, J., Mebane, L. M., Philippar, U., Pinheiro, E. M., Burds, A. A. et al. (2007). Ena/VASP Is Required for neuritogenesis in the developing cortex. *Neuron* 56, 441-455. 10.1016/j.neuron.2007.09.008 17988629

[JCS261527C33] Lebrand, C., Dent, E. W., Strasser, G. A., Lanier, L. M., Krause, M., Svitkina, T. M., Borisy, G. G. and Gertler, F. B. (2004). Critical role of Ena/VASP proteins for filopodia formation in neurons and in function downstream of netrin-1. *Neuron* 42, 37-49. 10.1016/S0896-6273(04)00108-4 15066263

[JCS261527C34] Lim, K. L., Chew, K. C. M., Tan, J. M. M., Wang, C., Chung, K. K. K., Zhang, Y., Tanaka, Y., Smith, W., Engelender, S., Ross, C. A. et al. (2005). Parkin mediates nonclassical, proteasomal-independent ubiquitination of synphilin-1: implications for Lewy body formation. *J. Neurosci.* 25, 2002-2009. 10.1523/JNEUROSCI.4474-04.2005 15728840 PMC6726069

[JCS261527C35] Lin, W.-H., Nebhan, C. A., Anderson, B. R. and Webb, D. J. (2010). Vasodilator-stimulated phosphoprotein (VASP) induces actin assembly in dendritic spines to promote their development and potentiate synaptic strength. *J. Biol. Chem.* 285, 36010-36020. 10.1074/jbc.M110.129841 20826790 PMC2975223

[JCS261527C36] Lin, S., Lu, S., Mulaj, M., Fang, B., Keeley, T., Wan, L., Hao, J., Muschol, M., Sun, J. and Yang, S. (2016). Monoubiquitination inhibits the actin bundling activity of Fascin. *J. Biol. Chem.* 291, 27323-27333. 10.1074/jbc.M116.767640 27879315 PMC5207158

[JCS261527C37] Lin, Z., Huang, J., Zhu, L., Lin, X., Huang, Y., Chen, C. and Pan, X. (2023). TRIM9 interacts with ZEB1 to suppress esophageal cancer by promoting ZEB1 protein degradation via the UPP pathway. *BioMed Res. Int.* 2023, 2942402. 37124931 10.1155/2023/2942402 PMC10139803

[JCS261527C38] Loureiro, J. J., Rubinson, D. A., Bear, J. E., Baltus, G. A., Kwiatkowski, A. V. and Gertler, F. B. (2002). Critical roles of phosphorylation and actin binding motifs, but not the central proline-rich region, for Ena/vasodilator-stimulated phosphoprotein (VASP) function during cell migration. *Mol. Biol. Cell* 13, 2533-2546. 10.1091/mbc.e01-10-0102 12134088 PMC117332

[JCS261527C39] Menon, S., Boyer, N. P., Winkle, C. C., McClain, L. M., Hanlin, C. C., Pandey, D., Rothenfußer, S., Taylor, A. M. and Gupton, S. L. (2015). The E3 ubiquitin ligase TRIM9 is a Filopodia off switch required for netrin-dependent axon guidance. *Dev. Cell* 35, 698-712. 10.1016/j.devcel.2015.11.022 26702829 PMC4707677

[JCS261527C41] Menon, S., Goldfarb, D., Ho, C. T., Cloer, E. W., Boyer, N. P., Hardie, C., Bock, A. J., Johnson, E. C., Anil, J., Major, M. B. et al. (2021). The TRIM9/TRIM67 neuronal interactome reveals novel activators of morphogenesis. *Mol. Biol. Cell* 32, 314-330. 10.1091/mbc.E20-10-0622 33378226 PMC8098814

[JCS261527C42] Mishima, C., Kagara, N., Matsui, S., Tanei, T., Naoi, Y., Shimoda, M., Shimomura, A., Shimazu, K., Kim, S. J. and Noguchi, S. (2015). Promoter methylation of TRIM9 as a marker for detection of circulating tumor DNA in breast cancer patients. *SpringerPlus* 4, 635. 10.1186/s40064-015-1423-7 26543769 PMC4627990

[JCS261527C43] Nakagawa, T. and Nakayama, K. (2015). Protein monoubiquitylation: targets and diverse functions. *Genes Cells* 20, 543-562. 10.1111/gtc.12250 26085183 PMC4744734

[JCS261527C44] Parker, S. S., Ly, K. T., Grant, A. D., Sweetland, J., Wang, A. M., Parker, J. D., Roman, M. R., Saboda, K., Roe, D. J., Padi, M. et al. (2023). EVL and MIM/MTSS1 regulate actin cytoskeletal remodeling to promote dendritic filopodia in neurons. *J. Cell Biol.* 222, e202106081. 10.1083/jcb.202106081 36828364 PMC9998662

[JCS261527C45] Perez-Riverol, Y., Xu, Q.-W., Wang, R., Uszkoreit, J., Griss, J., Sanchez, A., Reisinger, F., Csordas, A., Ternent, T., Del-Toro, N. et al. (2016). PRIDE inspector toolsuite: moving toward a universal visualization tool for proteomics data standard formats and quality assessment of ProteomeXchange datasets. *Mol. Cell. Proteomics* 15, 305-317. 10.1074/mcp.O115.050229 26545397 PMC4762524

[JCS261527C46] Perez-Riverol, Y., Bai, J., Bandla, C., García-Seisdedos, D., Hewapathirana, S., Kamatchinathan, S., Kundu, D. J., Prakash, A., Frericks-Zipper, A., Eisenacher, M. et al. (2022). The PRIDE database resources in 2022: a hub for mass spectrometry-based proteomics evidences. *Nucleic Acids Res.* 50, D543-D552. 10.1093/nar/gkab1038 34723319 PMC8728295

[JCS261527C47] Plooster, M., Menon, S., Winkle, C. C., Urbina, F. L., Monkiewicz, C., Phend, K. D., Weinberg, R. J. and Gupton, S. L. (2017). TRIM9-dependent ubiquitination of DCC constrains kinase signaling, exocytosis, and axon branching. *Mol. Biol. Cell* 28, 2374-2385. 10.1091/mbc.e16-08-0594 28701345 PMC5576901

[JCS261527C48] Pollard, T. D. and Cooper, J. A. (2009). Actin, a central player in cell shape and movement. *Science* 326, 1208-1212. 10.1126/science.1175862 19965462 PMC3677050

[JCS261527C49] Reinhard, M., Halbrügge, M., Scheer, U., Wiegand, C., Jockusch, B. M. and Walter, U. (1992). The 46/50 kDa phosphoprotein VASP purified from human platelets is a novel protein associated with actin filaments and focal contacts. *EMBO J.* 11, 2063-2070. 10.1002/j.1460-2075.1992.tb05264.x 1318192 PMC556672

[JCS261527C50] Riquelme, D. N., Meyer, A. S., Barzik, M., Keating, A. and Gertler, F. B. (2015). Selectivity in subunit composition of Ena/VASP tetramers. *Biosci. Rep.* 35, e00246. 10.1042/BSR20150149 26221026 PMC4721544

[JCS261527C51] Rolli-Derkinderen, M., Sauzeau, V., Boyer, L., Lemichez, E., Baron, C., Henrion, D., Loirand, G. and Pacaud, P. (2005). Phosphorylation of serine 188 protects RhoA from ubiquitin/proteasome-mediated degradation in vascular smooth muscle cells. *Circ. Res.* 96, 1152-1160. 10.1161/01.RES.0000170084.88780.ea 15890975

[JCS261527C52] Rottner, K., Behrendt, B., Small, J. V. and Wehland, J. (1999). VASP dynamics during lamellipodia protrusion. *Nat. Cell Biol.* 1, 321-322. 10.1038/13040 10559946

[JCS261527C53] Schindelin, J., Arganda-Carreras, I., Frise, E., Kaynig, V., Longair, M., Pietzsch, T., Preibisch, S., Rueden, C., Saalfeld, S., Schmid, B. et al. (2012). Fiji: an open-source platform for biological-image analysis. *Nat. Methods* 9, 676-682. 10.1038/nmeth.2019 22743772 PMC3855844

[JCS261527C54] Shekarabi, M. and Kennedy, T. E. (2002). The Netrin-1 receptor DCC promotes filopodia formation and cell spreading by activating Cdc42 and Rac1. *Mol. Cell. Neurosci.* 19, 1-17. 10.1006/mcne.2001.1075 11817894

[JCS261527C55] Skruber, K., Warp, P. V., Shklyarov, R., Thomas, J. D., Swanson, M. S., Henty-Ridilla, J. L., Read, T.-A. and Vitriol, E. A. (2020). Arp2/3 and Mena/VASP require profilin 1 for actin network assembly at the leading edge. *Curr. Biol.* 30, 2651-2664.e5. 10.1016/j.cub.2020.04.085 32470361 PMC7375932

[JCS261527C56] Smolenski, A., Bachmann, C., Reinhard, K., Hönig-Liedl, P., Jarchau, T., Hoschuetzky, H. and Walter, U. (1998). Analysis and regulation of vasodilator-stimulated phosphoprotein serine 239 phosphorylation in vitro and in intact cells using a phosphospecific monoclonal antibody*. *J. Biol. Chem.* 273, 20029-20035. 10.1074/jbc.273.32.20029 9685341

[JCS261527C57] Smolenski, A., Poller, W., Walter, U. and Lohmann, S. M. (2000). Regulation of human endothelial cell focal adhesion sites and migration by cGMP-dependent protein kinase I. *J. Biol. Chem.* 275, 25723-25732. 10.1074/jbc.M909632199 10851246

[JCS261527C71] Spudich, J. A. and Watt, S. (1971). The regulation of rabbit skeletal muscle contraction. I. Biochemical studies of the interaction of the tropomyosin-troponin complex with actin and the proteolytic fragments of myosin. *J. Biol. Chem.* 246, 4866-4871. 4254541

[JCS261527C58] Tanji, K., Kamitani, T., Mori, F., Kakita, A., Takahashi, H. and Wakabayashi, K. (2010). TRIM9, a novel brain-specific E3 ubiquitin ligase, is repressed in the brain of Parkinson's disease and dementia with Lewy bodies. *Neurobiol. Dis.* 38, 210-218. 10.1016/j.nbd.2010.01.007 20085810 PMC2942959

[JCS261527C59] Tokarz, D. A., Heffelfinger, A. K., Jima, D. D., Gerlach, J., Shah, R. N., Rodriguez–Nunez, I., Kortum, A. N., Fletcher, A. A., Nordone, S. K., Law, J. M. et al. (2017). Disruption of Trim9 function abrogates macrophage motility in vivo. *J. Leukoc. Biol.* 102, 1371-1380. 10.1189/jlb.1A0816-371R 29021367 PMC6608060

[JCS261527C60] van der Wal, L., Bezstarosti, K., Sap, K. A., Dekkers, D. H. W., Rijkers, E., Mientjes, E., Elgersma, Y. and Demmers, J. A. A. (2018). Improvement of ubiquitylation site detection by Orbitrap mass spectrometry. *J. Proteomics* 172, 49-56. 10.1016/j.jprot.2017.10.014 29122726

[JCS261527C61] Vignjevic, D., Kojima, S., Aratyn, Y., Danciu, O., Svitkina, T. and Borisy, G. G. (2006). Role of fascin in filopodial protrusion. *J. Cell Biol.* 174, 863-875. 10.1083/jcb.200603013 16966425 PMC2064340

[JCS261527C62] Wei, J., Mialki, R. K., Dong, S., Khoo, A., Mallampalli, R. K., Zhao, Y. and Zhao, J. (2013). A new mechanism of RhoA ubiquitination and degradation: roles of SCFFBXL19 E3 ligase and Erk2. *Biochim. Biophys. Acta* 1833, 2757-2764. 10.1016/j.bbamcr.2013.07.005 23871831 PMC3834026

[JCS261527C63] Winkle, C. C., Olsen, R. H. J., Kim, H., Moy, S. S., Song, J. and Gupton, S. L. (2016). Trim9 deletion alters the morphogenesis of developing and adult-born hippocampal neurons and impairs spatial learning and memory. *J. Neurosci.* 36, 4940-4958. 10.1523/JNEUROSCI.3876-15.2016 27147649 PMC4854964

[JCS261527C64] Yang, F., Liu, H., Yu, Y. and Xu, L. (2020). TRIM9 overexpression promotes uterine leiomyoma cell proliferation and inhibits cell apoptosis via NF-κB signaling pathway. *Life Sci.* 257, 118101. 10.1016/j.lfs.2020.118101 32679146

[JCS261527C65] Zeng, J., Wang, Y., Luo, Z., Chang, L.-C., Yoo, J. S., Yan, H., Choi, Y., Xie, X., Deverman, B. E., Gradinaru, V. et al. (2019). TRIM9-mediated resolution of neuroinflammation confers neuroprotection upon ischemic stroke in mice. *Cell Rep.* 27, 549-560.e6. 10.1016/j.celrep.2018.12.055 30970257 PMC6485958

[JCS261527C66] Zhang, Z.-C., Zhao, H.-F., Sun, Z., Li, Y., Zhong, M.-L., Wang, B.-H. and Jiang, X.-Z. (2023). Tripartite motif–containing 9 promoted proliferation and migration of bladder cancer cells through CEACAM6-Smad2/3 axis. *J. Cell Commun. Signal* 17, 1323-1333. 10.1007/s12079-023-00766-7 37249822 PMC10713968

[JCS261527C67] Zimmermann, D., Morganthaler, A. N., Kovar, D. R. and Suarez, C. (2016). In vitro biochemical characterization of cytokinesis actin-binding proteins. In *Yeast Cytokinesis: Methods and Protocols* (ed. A. Sanchez-Diaz and P. Perez), pp. 151-179. New York, NY: Springer. 10.1007/978-1-4939-3145-3_12 26519312

[JCS261527C68] Zuzga, D. S., Pelta-Heller, J., Li, P., Bombonati, A., Waldman, S. A. and Pitari, G. M. (2012). Phosphorylation of vasodilator-stimulated phosphoprotein Ser239 suppresses Filopodia and Invadopodia in colon cancer. *Int. J. Cancer* 130, 2539-2548. 10.1002/ijc.26257 21702043 PMC3236815

