# Supplementary material for: Multi-monoubiquitylation controls VASP-mediated actin dynamics
Source: J Cell Sci. 2024 Jan 29;137(2):jcs261527. doi: 10.1242/jcs.261527 (PMC10917064; doi:10.1242/jcs.261527)
Supplement: Supplementary information [file joces-137-261527-s1.pdf]

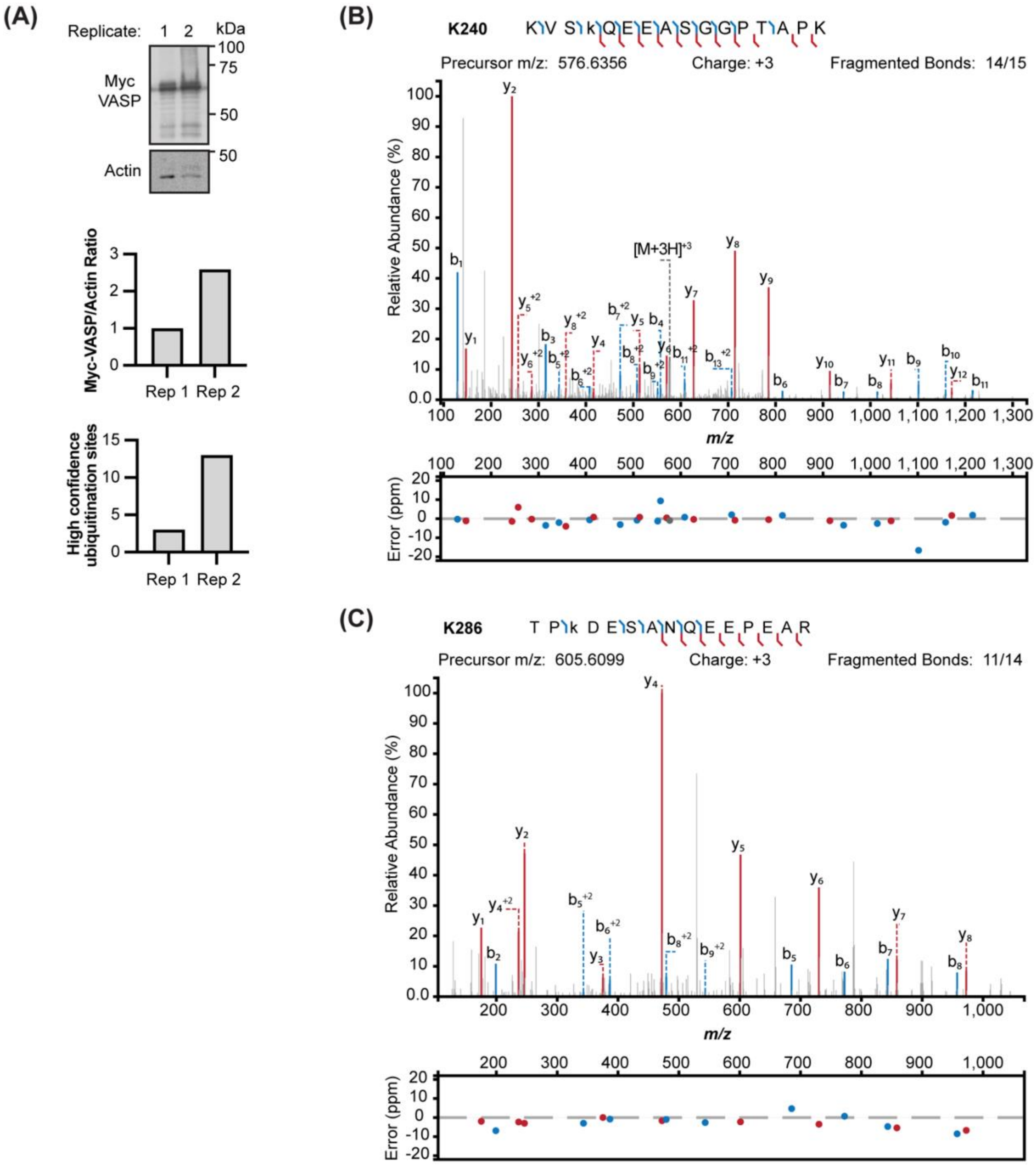

Fig. S1. MS/MS Spectra of ubiquitinated peptides. (A) Western blotting of Myc-VASP inputs for each proteomic experiment. The ratio of Myc-VASP to actin was calculated for each replicate. (B) MS/MS spectrum of the triply charged ion (m/z 576.9678) corresponding to VASP tryptic peptide KVSKQEEASGGPTAPK. K4 is diGly modified (k), corresponding to K240 in VASP. (C) MS/MS spectrum of the triply charged ion (m/z 605.6099) corresponding to VASP tryptic peptide TPKDESANQEEPEAR. K3 is diGly modified (k), corresponding to K286 in VASP.

Journal of Cell Science • Supplementary information

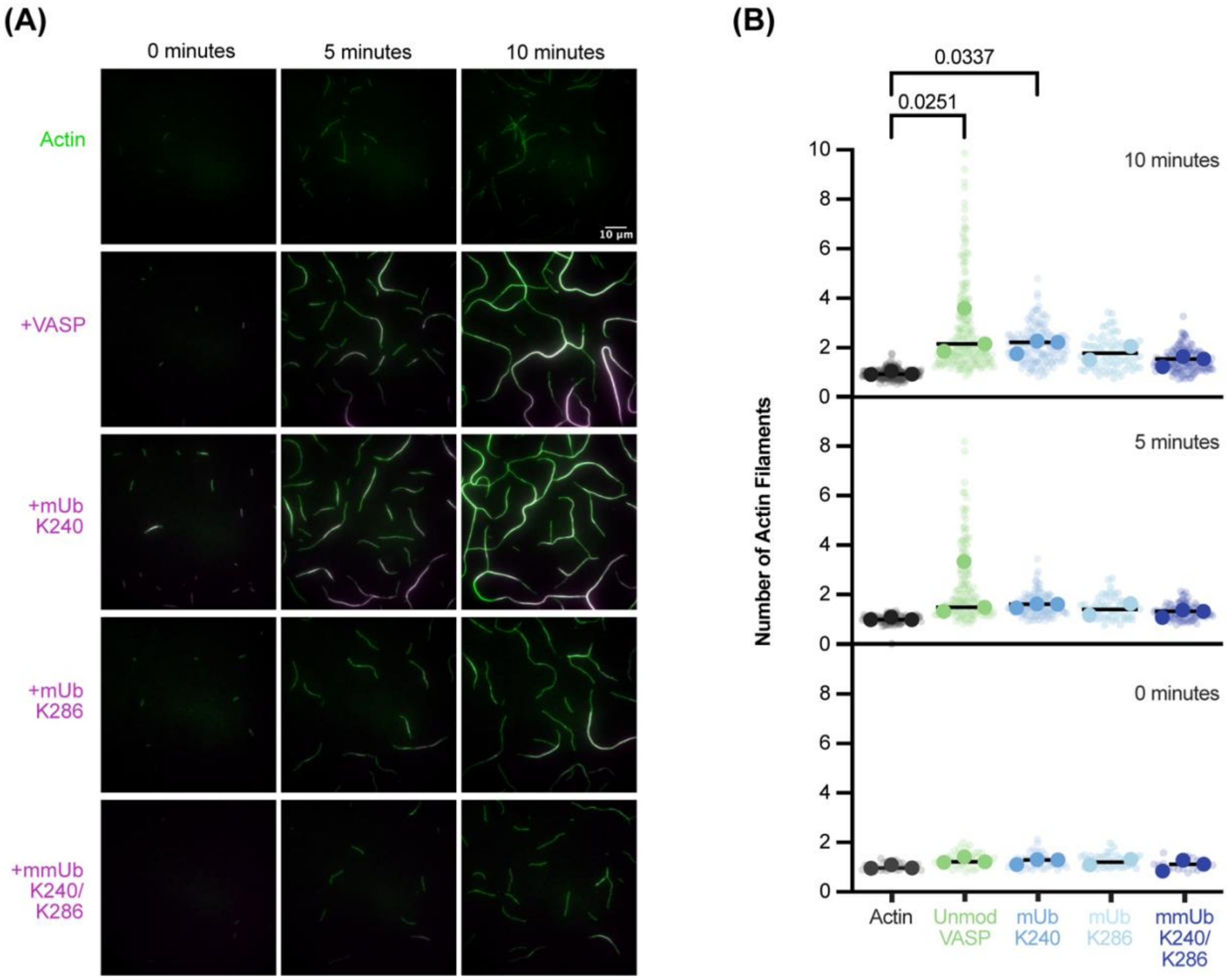

Fig. S2. mUb-K286 and mmUb-K240/K286 display decreased actin bundling activity. (A) Polymerization of 1.5 µM actin (10% AlexaFluor-488 labeled, shown in green) visualized with TIRF microscopy for ten minutes in the presence of 25 nM TAMRA-VASP (shown in magenta). Full time-lapse imaging is shown in Movie 1. Scale bar, 10 µm. (B) Number of actin filaments per bundle, based on the fluorescence intensity of AF-488 actin. Data was quantified from TIRF microscopy movies displayed in Fig 3D at 0, 5, and 10 minutes. N = 2-3 independent experiments encompassing 28-41, 55-147, and 73-220 filaments/bundles for the 0, 5, and 10 minute timepoints respectively. Lines represent median number of actin filaments. Statistical significance calculated with the Kruskal-Wallis test and Dunn's multiple comparison test.

Journal of Cell Science • Supplementary information

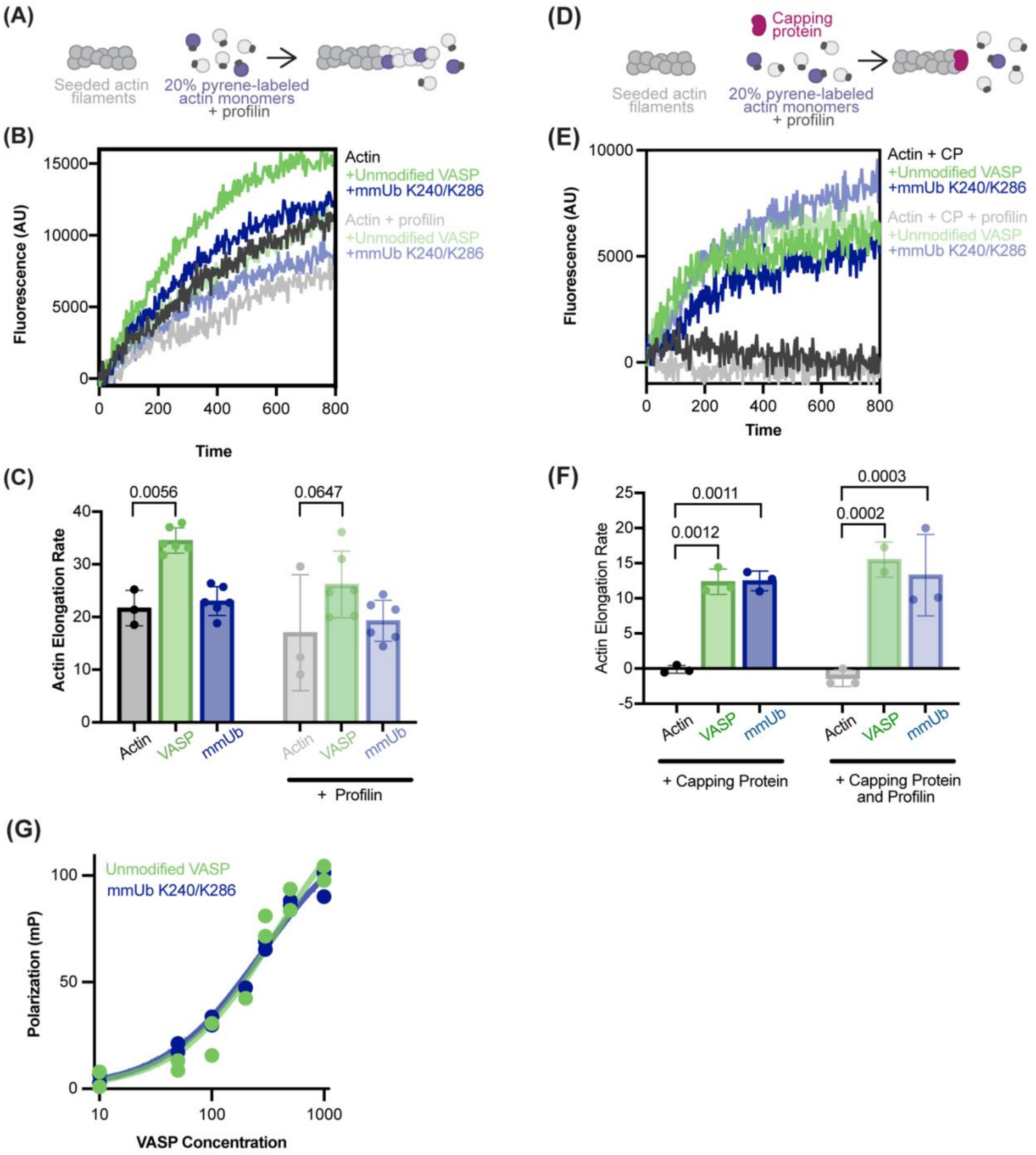

Fig. S3. mmUb-K240/K286 maintains actin monomer and profilin-actin binding.
(A) Schematic of pyrene actin assays in the presence of profilin. (B) Fluorescent traces of 0.5 µM (20% pyrene-labeled) actin monomer elongation from preformed actin seeds (1 µM) in the presence of 5 µM profilin and 40 nM VASP. (C) The initial elongation rate

Journal of Cell Science • Supplementary information

from seeded pyrene elongation assays in the presence of 5 µM profilin (calculated from the first 300 s of the assay). N = 3-6 measurements collected across 3 independent experiments. Bar represents mean with standard deviation. Statistics were calculated with a one-way ANOVA with Sidak's multiple comparisons. (D) Schematic of pyrene actin assays in the presence of profilin and capping protein. (E) Fluorescent traces of 0.5 µM (20% pyrene-labeled) actin monomer elongation from preformed actin seeds (1 µM) in the presence of 1 µM profilin, 5 nM capping protein, and 40 nM VASP. (F) The initial elongation rate from seeded pyrene elongation assays in the presence of 5 µM profilin (calculated from the first 300 s of the assay). N = 2-3 measurements collected across 3 independent experiments. Bar represents mean with standard deviation. Statistics were calculated with a one-way ANOVA with Sidak's multiple comparisons. (G) Fluorescence polarization of 40nM AF488-Actin incubated with varying concentrations of VASP for 30 minutes.

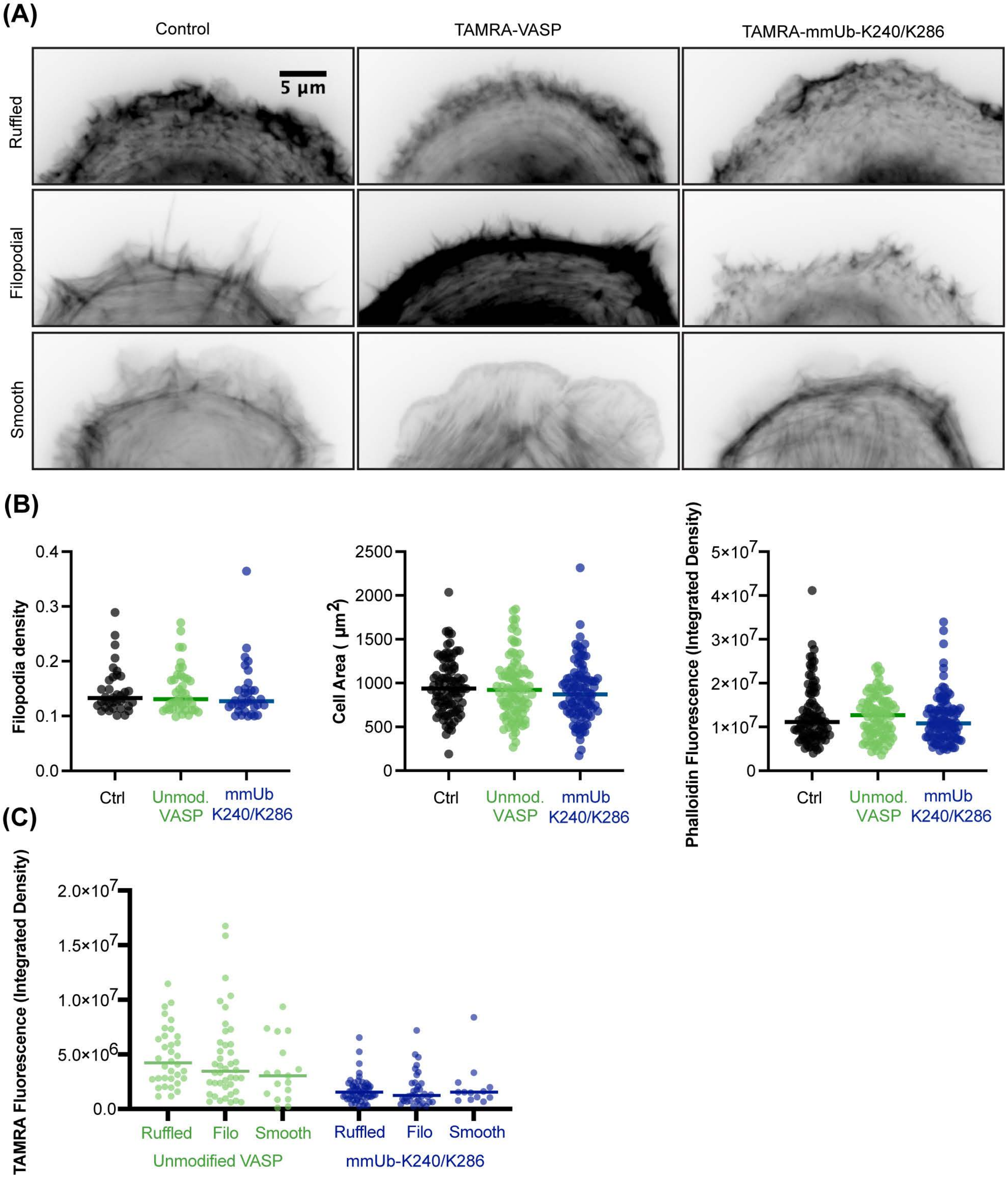

Fig. S4. Electroporation of mmUb-K240/K286 does not change filopodial density, cell area or phalloidin-stained actin levels. (A) Representative widefield phalloidin images of each cell spreading phenotype. (B) Quantification of filopodia density, cell area, and phalloidin fluorescence intensity. 92-104 cells were quantified for each condition across three experiments. Line represents the median of all data points. (C) TAMRA fluorescence intensity separated by cell spreading phenotype. N = 13-54 cells per classification across three experiments. Line represents the median of all data points.

Journal of Cell Science • Supplementary information

Table S1. Curve-fitting parameters for actin pyrene elongation assays.

	Unmodified	mUb-K240	mUb-K286	mmUb-K240/K286
B_{max}	45.76	31.66	41.86	17.71
B_{max} – 95% CI	37.62-57.65	22.25-48.80	21.92 to +infinity	7.533 to +infinity
K_D^{app} (nM)	25.45	17.99	43.75	54.89
K_D^{app} (nM) – 95% CI	14.24-46.30	5.347-58.12	5.932- +infinity	5.122- +infinity

Table S2. Curve-fitting parameters for actin pyrene elongation assays in the presence of capping protein.

	Unmodified	mUb-K240	mUb-K286	mmUb-K240/K286
B_{max}	33.56	28.55	33.93	27.04
B_{max} – 95% CI	27.59-41.15	23.61-34.58	28.17-41.17	22.36-32.98
K_D^{app} (nM)	37.83	24.88	36.19	42.88
K_D^{app} (nM) – 95% CI	18.18-71.54	10.60-49.18	17.74-67.02	21.43-78.37

Journal of Cell Science • Supplementary information

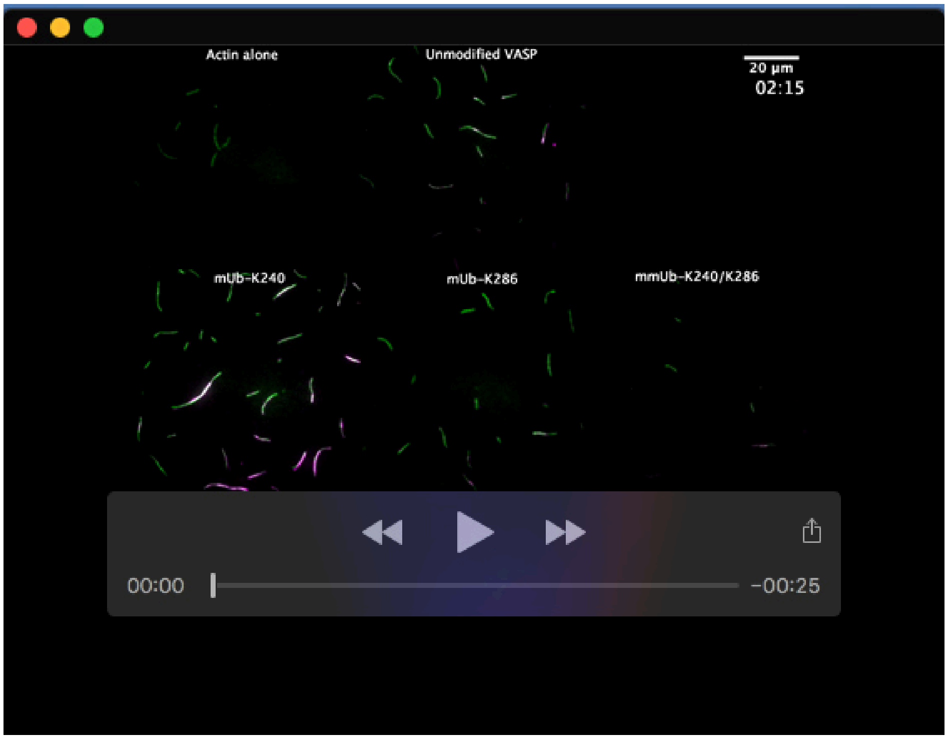

Movie 1. Polymerization of 1.5 µM actin (10% AlexaFluor-488 labeled, shown in green) visualized with TIRF microscopy for ten minutes (2.5 s acquisition time) in the presence of 25 nM TAMRA-VASP (shown in magenta). Frame rate 30 fps.

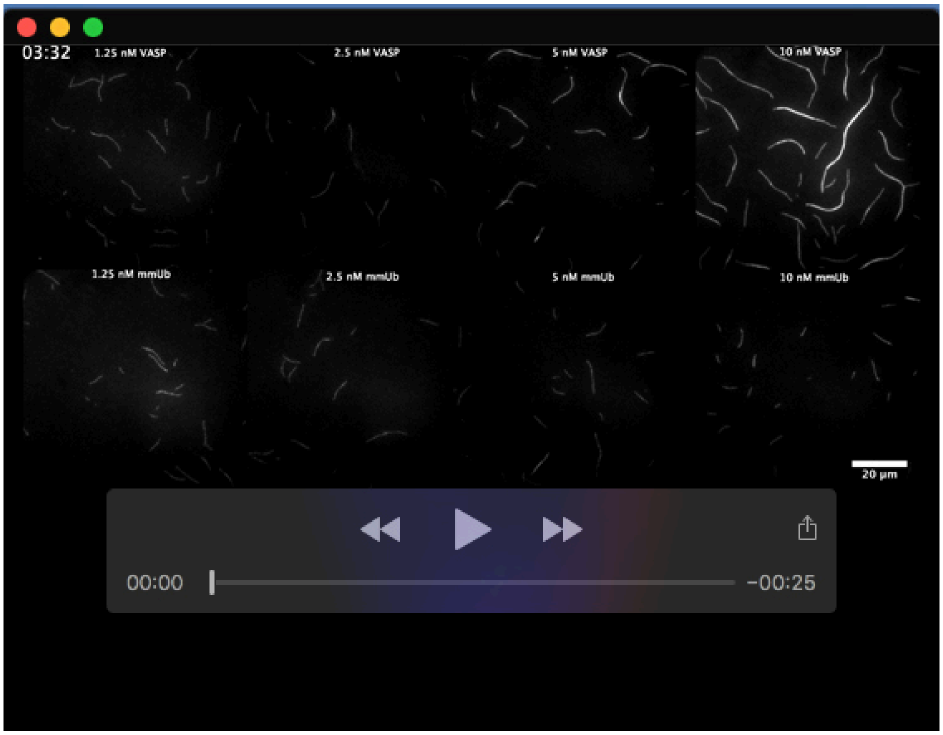

Movie 2. Polymerization of 1.5 µM actin (10% AlexaFluor-488 labeled) visualized with TIRF microscopy for ten minutes (2.5 s acquisition time) in the presence of 1.25-10 nM TAMRA-VASP (not shown). Frame rate 30 fps.

Blot Transparency
Figure 1A

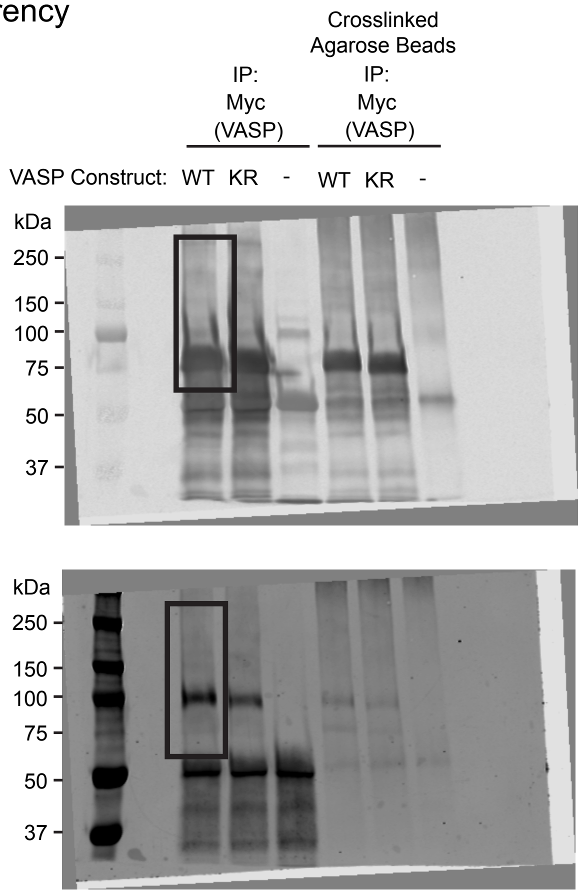

Myc
(VASP)

Antibody: α-Myc
9E10 Hybridoma serum
1:2000 WB

HA
(Ub)

Antibody: α-HA
ThermoFisher Scientific Cat #71-5500
1:250 WB

Figure S1A

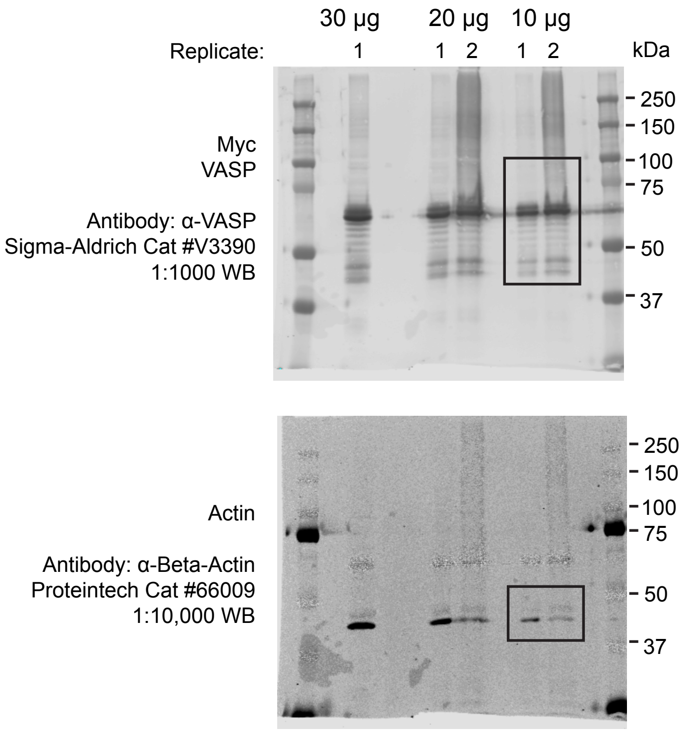

Fig. S5. Blot Transparency

Journal of Cell Science • Supplementary information
