# Supplementary material for: Multi-monoubiquitylation controls VASP-mediated actin dynamics
Source: J Cell Sci. 2024 Jan 29;137(2):jcs261527. doi: 10.1242/jcs.261527 (PMC10917064; doi:10.1242/jcs.261527)
Supplement: Supplementary file 1 [file joces-137-261527_review_history.pdf]

Multi-monoubiquitylation controls VASP-mediated actin dynamics

Laura E. McCormick, Cristian Suarez, Laura E. Herring, Kevin S Cannon, David R. Kovar,
Nichols G. Brown and Stephanie L. Gupton
DOI: 10.1242/jcs.261527

Editor: Michael Way

Review timeline
Original submission: 28 July 2023
Editorial decision: 13 September 2023
First revision received: 21 November 2023
Accepted: 13 December 2023

Original submission

First decision letter

MS ID#: JOCES/2023/261527

MS TITLE: Multi-monoubiquitination controls VASP-mediated actin dynamics

AUTHORS: Laura E McCormick, Cristian Suarez, Laura E Herring, Kevin S Cannon, David R Kovar, Nicholas G Brown, and Stephanie Gupton
ARTICLE TYPE: Research Article

We have now reached a decision on the above manuscript.

To see the reviewers' reports and a copy of this decision letter, please go to: https://submit-jcs.biologists.org and click on the 'Manuscripts with Decisions' queue in the Author Area. (Corresponding author only has access to reviews.)

As you will see, all three reviewers were positive but raise a number of criticisms and points of clarification that prevent me from accepting the paper at this stage. They suggest, however, that a revised version might prove acceptable, if you can address their concerns. If you think that you can deal satisfactorily with the criticisms on revision, I would be pleased to see a revised manuscript. We would then return it to the reviewers.

Please ensure that you clearly highlight all changes made in the revised manuscript. Please avoid using 'Tracked changes' in Word files as these are lost in PDF conversion.

I should be grateful if you would also provide a point-by-point response detailing how you have dealt with the points raised by the reviewers in the 'Response to Reviewers' box. Please attend to all of the reviewers' comments. If you do not agree with any of their criticisms or suggestions please explain clearly why this is so.

Reviewer 1

Advance summary and potential significance to field

The E3 ligase TRIM9 has previously been seen to colocalise with and ubiquitinate VASP. This activity is proposed to be a controllable step in filopodial regulation because it responds to the guidance

© 2023. Published by The Company of Biologists under the terms of the Creative Commons Attribution License (https://creativecommons.org/licenses/by/4.0/).

cue netrin that tunes filopodia number and stability in neurons. Finding out how signals control actin activity and downstream cytoskeletal morphology is key for understanding the molecular basis of neuronal migration and morphology and is important because of the role of these processes in neurodevelopmental disorders (autism, intellectual disability etc).

Reducing the levels of Ena/VASP proteins reduces filopodia, although other actin elongators are also involved. VASP itself binds the barbed ends of actin filaments, competes with capping protein and also interacts/bundles actin filaments laterally. VASP increases the polymerisation rate of the actin filament which is further increased when there are multiple VASPs or multiple actin filaments. VASP is also regulated by phosphorylation and is a tetramer with G and F-binding domains which are thought to mediate its activity on actin filaments and in turn, on filopodia.

In this manuscript, McCormick at al have identified two main ubiquitination sites on VASP, in the G and F actin binding regions and engineered proteins with ubiquitination via a cysteine linkage and assayed the mono or multiple mono-ubiquinated VASP for effects on the various actin properties. They evaluated VASP oligomerisation, bulk actin polymerisation and bundling, single filament and bundle elongation, anti-capping activity, barbed end binding and in cells enrichment to filopodia and in influencing cell morphology. They have determined that the major effects of ubiquitination are in filament elongation in vitro and localisation to filopodia and other actin-rich sites in cells. In the discussion they describe a model of ubiquitination affecting the mobility of VASP regions in adding actin subunits to actin filaments.

I found the paper enjoyable and compelling. The levels of VASP and the mono and multi/mono-ubiquitinated forms have been carefully titrated, repeated and quantified and all credit is due for this careful work. The increase in actin filament elongation rate by VASP compared with native actin elongation alone is relatively modest (~2 fold) so detecting a difference is not so easy.

Comments for the author

Suggestions to authors

For the TIRFM actin filament assays, I found Video 2 interesting. At 1.25 nM VASP the single filament polymerisation is similar, the difference really comes with the higher concentrations of VASP which is when the actin filaments bundle. The authors say this in their text (pg 7) although I felt the defect in bundling in the mmUb protein wasn't coming through in their quantifications. I wonder if the authors have considered quantifying the actin and VASP fluorescence intensities or taking ratios between them from these videos?

The conclusion is that 'enhancement of new actin monomer addition is impaired by multi monoubiquitination of VASP', are the authors thinking this is both in cis on a given filament and in trans across filaments? Thinking structurally it would give a different picture on the type of mobility.

In the anti-capping measurements, both mmUb or native VASP proteins are effective, although there's a shift in the curve in Fig 4C and the concentration at which the curves plateau is ~70 nM for mono-Ub and native compared with 200 nM for mmUb-VASP, which again suggests that there's a multimeric/bundling deficiency in the mmUb-VASP. Referring to the quantified curve fits in Table 2 it doesn't look like the same data – might be worthwhile double checking if something went awry in either the graph or table. It could be the differences aren't significant.

In the text, the authors comment on measuring the fascin-actin dwell time because it's 2-4 s depending on filament numbers, rather than 1 s (page 9). However the numbers for dwell times don't seem to correspond to this because they are 1.1 for both native and mmUb VASP. The legend of Fig 4F doesn't mention fascin. I wonder if there's a text error. The shorter timescale or description of the experiment needs explaining or correcting.

The use of electroporation of the Ub conjugated protein seemed to work well and the quantifications look reproducible. I found the comparison in intensities between GFP-VASP and the TAMRA VASP convincing given the inherent constraints in evaluating ubiquitination as a modification. I would be interested to see the quantifications of VASP and mmUb-VASP at the focal

© 2023. Published by The Company of Biologists under the terms of the Creative Commons Attribution License (https://creativecommons.org/licenses/by/4.0/).

adhesions as in Panel A it looks strikingly reduced. I wasn't wholly convinced that mmUb-VASP would be a filopodia specific regulation (as in the last sentence), so it would be interesting for a bit more reasoning and the focal adhesion quantification.

Altogether I felt the manuscript gives strong support that multimonoubiquitination reduces VASP actin elongation and localisation to actin structures in cells and thus is a point of inhibitory intervention in regulating actin dynamics.

Minor comments

Fig 2E panel it would be helpful to have 'VASP' added to "mUb" and mmUb to say 'mUb-VASP' etc. Typo last line of the Fig 2 legend, an 'm' is missing I think as it's the mmUb condition.

Fig 3C panel it would be helpful to have 'CP' on there and in the legend. In the present copy the text is a couple of pages away from the legend so it helps to refer back to the correct graph.

Fig 3E graph axis, 25 and 12.5 do not make for easy reading off the sub-ticks on the axis, could you alter it to 12 and 24? Then each subtick is 4!

I felt the conclusions could be better focused on this manuscript. On page 9, the link to cancer and ubiquitination of Ras I felt was not so relevant, I also felt the paragraph 'Deciphering the ubiquitination code' didn't really add anything.

Reviewer 2

Advance summary and potential significance to field

Members of the Ena/VASP protein family have been implicated in assembly of actin filaments in cellular structures like filopodia which contain linear actin networks. In a previous study, authors showed that reversible non-degradative ubiquitination regulates the function of VASP in neuronal cells. However, how these modifications influence actin-related activities of VASP has so far remained unresolved. In the current study, the authors employ a number of in vitro biochemical and cell biological assays to investigate how multi-monoubiquitination affects VASP's actin-related activities. This study is timely, and valuable to the field.

Comments for the author

Members of the Ena/VASP protein family have been implicated in assembly of actin filaments in cellular structures like filopodia which contain linear actin networks. In a previous study, authors showed that reversible non-degradative ubiquitination regulates the function of VASP in neuronal cells. However, how these modifications influence actin-related activities of VASP has so far remained unresolved. In the current study, the authors employ a number of in vitro biochemical and cell biological assays to investigate how multi-monoubiquitination affects VASP's actin-related activities. This study is timely, and valuable to the field.
Nevertheless, I feel the differences in activities of unmodified VASP and multi-monoubiquitinated VASP are modest at best. This is especially true for their in vitro results (fig. 3 and 4). To their credit, the authors themselves state "collectively, pyrene elongation assays (both in the presence and absence of capping protein) and single molecule TIRFM assays suggest similar barbed end binding dynamics for unmodified VASP and mmUb-K240/K286". As a result, their in vitro results don't strongly seem to explain their cellular observations which seem more convincing.

A few issues that might help authors improve the manuscript.
1. In the last paragraph of introduction, authors state we demonstrate that mimicking multi-monoubiquitination of VASP reduces actin filament binding and bundling". The results presented in Figure 4f however show the opposite – multi-monoubiquitination of VASP doesn't change barbed end binding as evidenced by unchanged barbed end dwell times. Are they suggesting, that the effects are solely from VASP's binding to filament sides? Further, do the experiments in Fig. 3D confirm their conclusions about monoubiquitination reducing bundling?

© 2023. Published by The Company of Biologists under the terms of the Creative Commons Attribution License (https://creativecommons.org/licenses/by/4.0/).

2. I am a bit confused was to why the mass of the double ubiquitinated species in Figure 1E (bottom plot) isn't higher than the mono-ubiquitinated species (2nd and 3rd plot from the top)? The average mass of the multi ubiquitinated protein actually appears to be lower than what was seen for mono-ubiquitinated species.
This is all the more perplexing since majority of VASP appears to be multi- ubiquitinated in the Coomassie gel shown in Fig. 1d. May be I am missing something here. Can authors clarify why this is the case?
3. Fig. 3d. The contrast/intensity of the images could be improved (especially for those who read printed manuscripts). At first look, it appears that there are no filaments in the actin alone condition (top row). However this is not the case. The filaments are just very dim.
4. In Fig. 3D, I assume VASP is fluorescently labelled. Please mention this in the figure legend. It will also be helpful to expressly describe what the authors learn from the double-color TIRF experiments here. Can they use the data here to get an independent measure of VASP's side binding and bundling activities (see comment 1 above).
5. Authors (to their credit) note the conclusions from the pyrene assays in Fig. 3b don't entirely match their results of the TIRF assays. In pyrene assays, all 4 VASP conditions (unmodified VASP, mUB-240, mUB-286, mmUB-240/386) show higher polymerization than the condition with actin seeds. Due to addition of seeds, we can safely assume that the increase in pyrene signal is chiefly due to elongation and not nucleation. The authors "account this disparity to the higher sensitivity of the TIRF assays". More clarification is required here.
6. Further in Fig. 3f, I am uncertain that any strong conclusions can be derived from the data presented here. At 1.25 nM, monoubiquitinated VASP is slightly faster than unmodified VASP; at 2.5 nM, monoubiquitinated VASP is >20% slower than unmodified VASP; and at 5 nM, monoubiquitinated VASP is almost as fast as unmodified VASP. So I am not sure if just based on 10 nM concentration, authors can conclude that ubiquitination impairs elongation by VASP.
7. The role of profilin in VASP-mediated actin elongation as remained in debate. I am therefore unconvinced of authors' election to not test the effect of profilin in their experiments in Figure 3 and 4. I think it might help test if ubiquitin-modifications might influence VASP's activities in presence of profilin, especially since the effects in absence of profilin arent striking.
8.This is a minor point - in Fig. 4a, red spheres are supposed to denote capping protein. From the schematic, it appears two separate CP molecules can simultaneously bind barbed ends of an actin filament (2 red spheres occupy the barbed end of the actin filament). For sake of clarity, I suggest they adopt a different shape to denote CP such that only one molecule is bound at the barbed end at a time.
9.By the heading "Conclusions", I assume they meant "Discussion". I found this section long and difficult to follow, I believe this section could be presented in a more concise manner. Also a more direct discussion about how their in vitro results explain (or not explain) their cellular results will be helpful.

Reviewer 3

Advance summary and potential significance to field

Regulation of actin dynamics by post-translational (PTM) modification is an evolving field of studies which is of great importance. It becomes more and more evident that functions of cytoskeletal proteins themselves as well as their interacting partners are regulated by PTMs. In many cases, the functional role of these PTMs is unknown and/or mechanistic understanding of their effects is lacking. In this study the authors investigate the effects of ubiquitination of VASP on its regulatory role in regulation of actin dynamics. I enjoyed reading this paper – I think it's clearly written and presents novel mechanistic insights related to VASP regulation by ubiquitination (Ub). I appreciated the authors' creative approach to methodology (use of crosslinking, labeled peptide's fusion, TIRFM, etc.) which I found well justified and informative. Below I summarize the points which the authors should address/discuss before the paper is published.

Comments for the author

Main points:
1. The description of the mass spec and transfection experiments requires clarification.

© 2023. Published by The Company of Biologists under the terms of the Creative Commons Attribution License (https://creativecommons.org/licenses/by/4.0/).

Fig. 1B is unclear. Can the authors indicate (color-code?) the Ub sites ID-ed in two different experiments? Were all the additional Ub sites detected in only one of the two samples or each of them ID-ed some additional unique sites? I am puzzled by so many potential sites of ubiqitination. Could it be an artifact of iodoacetamide treatment?
Nielsen M. L., Vermeulen M., Bonaldi T., Cox J., Moroder L., Mann M. (2008) Iodoacetamide-induced artifact mimics ubiquitination in mass spectrometry. Nat. Methods 5, 459–460 Exploration of the Ub modifications of the two most prominent sites is justified, however, the rationale for testing multi-Ub is not very clear to me. With so many potential Ub sites it's not obvious which mmUb species would be present in cells. Based on the data presented, ~10kDa shift in VASP protein population was consistent with a single Ub modification (potentially collective contribution of Ub on different single sites) but no prominent band corresponding to multi-Ub was detected. The authors should reconcile this.
Overall level of the VASP expression (endogenous+exdogenous) would be useful to know. Having "mock" condition (Lipofectamine only) shown side-by-side with the transfected samples would be helpful assuming that reliable VASP Ab are available. Is it possible that overexpression of VASP triggers the Ub at multiple sites? Some of these sites are regulatory but some of them could later target misfolded protein for degradation. Could lowering the expression level of exdogenous VASP change the number of Ub detected?
2. I think that the use of cross-linking was an elegant and justified approach. However, the authors should comment in the Results or Discussion on the potential limitations of using BMOE (which has a spacer arm length of ~8A) vs covalent bond formed between VASP and Ub under cellular conditions. Potentially, Ub attached via BMOE may have more motional freedom which (potentially) could contribute to the observed effects.
3. Tables 2, 3 should be referred as Tables S1 and S2 in the main text. Additionally, I'd argue that fitting of the bulk pyrene assembly data might not yield true Kd values because of the combined contribution of actin nucleation, elongation, and (potentially) bundling to the signal. Additionally, the side-binding of VASP would change [VASP]free as actin polymerizes. Thus, I think it would be more accurate to use a different term such as Kd(app).
4. Fig. 3D. I wonder if performing the analysis of the bundles' thickness using the movies which the team currently has would help better characterization the effects of Ub modifications.
5. I suggest that the authors edit the Conclusions section. I think it would benefit from being more focused and from discussing (or commenting on) the limitations of the chosen approaches (see my comments 1-4).
Minor points:
I suggest rewording: "…Purple residues were selected for follow-up study…" (Fig. 1, legend)
Typo, Page 9. – "enrichment at each of these structures appeared dimin-ished"

First revision

<u>Author response to reviewers' comments</u>

<u>REVIEWER 1</u>

Comment 1: For the TIRFM actin filament assays, I found Video 2 interesting. At 1.25 nM VASP the single filament polymerisation is similar, the difference really comes with the higher concentrations of VASP which is when the actin filaments bundle. The authors say this in their text (pg 7) although I felt the defect in bundling in the mmUb protein wasn't coming through in their quantifications. I wonder if the authors have considered quantifying the actin and VASP fluorescence intensities or taking ratios between them from these videos?

Response:

Thank you for this suggestion. We have quantified the actin bundling of each VASP construct at 25 nM to examine bundling more closely in Figure S2. These new analysis are described in the results as such:

© 2023. Published by The Company of Biologists under the terms of the Creative Commons Attribution License (https://creativecommons.org/licenses/by/4.0/).

"Actin filaments/bundles were traced to measure the mean intensity of actin along the structure to approximate bundling over time. We observed a significant increase in the mean fluorescence intensity of filaments with both unmodified VASP and mUb-K240 at ten minutes. However, no significant change was observed for filaments with mUb-K286 and mmUb-K240/K286, suggesting decreased bundling activity (**Fig S2**)."

Comment 2: The conclusion is that 'enhancement of new actin monomer addition is impaired by multimonoubiquitination of VASP', are the authors thinking this is both in cis on a given filament and in trans across filaments? Thinking structurally it would give a different picture on the type of mobility.

Response:

This is an interesting point as VASP does function as a tetramer. In our TIRF movies, we quantified both single filament elongation rates and parallel actin bundles (which share similar elongation rates), suggesting that this would be cis. We have clarified in the text that we see these impacts of VASP on single actin filaments to emphasize this.

Comment 3: In the anti-capping measurements, both mmUb or native VASP proteins are effective, although there's a shift in the curve in Fig 4C and the concentration at which the curves plateau is ~70 nM for mono-Ub and native compared with 200 nM for mmUb-VASP, which again suggests that there's a multimeric/bundling deficiency in the mmUb-VASP. Referring to the quantified curve fits in Table 2 it doesn't look like the same data - might be worthwhile double checking if something went awry in either the graph or table. It could be the differences aren't significant.

Response: We have confirmed that these numbers in Table 2 are correct. The large 95% confidence interval does somewhat occlude the differences observed in the curve fits of Fig 4C.

Comment 4: In the text, the authors comment on measuring the fascin-actin dwell time because it's 2-4 s depending on filament numbers, rather than 1 s (page 9). However the numbers for dwell times don't seem to correspond to this because they are 1.1 for both native and mmUb VASP. The legend of Fig 4F doesn't mention fascin. I wonder if there's a text error. The shorter timescale or description of the experiment needs explaining or correcting.

Response: Figure 4D-F was completed in the presence of 673 nM fascin. We apologize for the ambiguity and have clarified this detail in the figure legend. Although Harker et al. calculated a dwell time of ~2.6 s for a 2 filament bundle, we hypothesize that differences in buffer compositions, the specific actin prep, and various other experimental changes may account for the reduced dwell time. The Kovar lab performs these assays regularly and have advised that this variability is within reason, in their experience.

Comment 5: The use of electroporation of the Ub conjugated protein seemed to work well and the quantifications look reproducible. I found the comparison in intensities between GFP-VASP and the TAMRA VASP convincing given the inherent constraints in evaluating ubiquitination as a modification. I would be interested to see the quantifications of VASP and mmUb-VASP at the focal adhesions as in Panel A it looks strikingly reduced. I wasn't wholly convinced that mmUb-VASP would be a filopodia specific regulation (as in the last sentence), so it would be interesting for a bit more reasoning and the focal adhesion quantification.

Response: Thank you for this comment. We did not intend to imply VASP ubiquitination was a filopodia specific regulation—rather, we often to refer to filopodia phenotypes due to our lab's previous work on growth cone filopodia and the colocalization of VASP with the E3 ubiquitin ligase TRIM9 at filopodia tips.

We do hypothesize that that VASP ubiquitination may certainly regulate other actin-based structures, as shown by our inclusion of the lamellipodia enrichment data. We have also updated the manuscript to demonstrating that mmUb-VASP also shows decreased enrichment at focal adhesions (Fig 6B).

© 2023. Published by The Company of Biologists under the terms of the Creative Commons Attribution License (https://creativecommons.org/licenses/by/4.0/).

Initially we intended to reflect our results back to our results in the growth cone, but we must not ignore the potential implications in non-neuronal cells and other actin structures. We have also revised this conclusion in the discussion in several places, including:

"Our in vitro results suggested that the ability of VASP to bind to the sides of actin filaments, bundle actin, and enhance filament elongation is impaired by multi-monoubiquitination. Intracellular VASP recruitment is strongly regulated by binding partners of the FPPPP motif within the EVH1 domain —such as lamellipodin, zyxin, and vinculin (Krause et al., 2004; Brindle et al., 1996; Ball et al., 2000). Additional papers have suggested that the actin binding activity of VASP may influence localization as well (Benz et al., 2009; Smolenski et al., 2000; Loureiro et al., 2002). The reduced actin side-binding activity of mmUb-VASP may account for the reduced localization of the ubiquitinated protein to actin-based structures such as the lamellipodia and focal adhesions. Furthermore, the reduced ability to enhance polymerization of actin filaments may impact the filopodial localization of mmUb-VASP and reduce the filopodial phenotype in actively spreading cells."

And:

"Collectively, TRIM9-mediated ubiquitination may serve as a filopodial-specific negative regulator of cytoskeletal remodeling in developing neurons. However, our new work in MV^{D7} cells suggests that VASP ubiquitination may also regulate actin dynamics at lamellipodia and focal adhesions."

Comment 6: Altogether I felt the manuscript gives strong support that multimonoubiquitination reduces VASP actin elongation and localisation to actin structures in cells and thus is a point of inhibitory intervention in regulating actin dynamics.

Thank you! We agree and are excited about this reversible regulation of VASP function.

Comment 7: Fig 2E panel it would be helpful to have 'VASP' added to "mUb" and mmUb to say 'mUb-VASP' etc. Typo last line of the Fig 2 legend, an 'm' is missing I think as it's the mmUb condition.

Response: We have updated Fig 2E and updated this typo in the Fig 2F legend.

Comment 8: Fig 3C panel it would be helpful to have 'CP' on there and in the legend. In the present copy the text is a couple of pages away from the legend so it helps to refer back to the correct graph.

Response: We have updated the y axis label of Fig 4C as well as the figure legend.

Comment 9: Fig 3E graph axis, 25 and 12.5 do not make for easy reading off the sub-ticks on the axis, could you alter it to 12 and 24? Then each subtick is 4!

Response: We have changed the subtick division in Fig E-F based on this suggestion.

Comment 10: I felt the conclusions could be better focused on this manuscript. On page 9, the link to cancer and ubiquitination of Ras I felt was not so relevant, I also felt the paragraph 'Deciphering the ubiquitination code' didn't really add anything.

Response: We have removed both of these sections from the discussion section. We have edited and streamlined the remaining discussion section for ease of reading.

REVIEWER 2

Comment 1: In the last paragraph of introduction, authors state we demonstrate that mimicking multi-monoubiquitination of VASP reduces actin filament binding and bundling". The results presented in Figure 4f however show the opposite – multi-monoubiquitination of VASP doesn't change barbed end binding as evidenced by unchanged barbed end dwell times. Are they

© 2023. Published by The Company of Biologists under the terms of the Creative Commons Attribution License (https://creativecommons.org/licenses/by/4.0/).

suggesting, that the effects are solely from VASP's binding to filament sides? Further, do the experiments in Fig. 3D confirm their conclusions about monoubiquitination reducing bundling?

Response:

In the specific introduction sentence that the reviewer has highlighted above, we are referring to filament side-binding (Fig 2A-C). We have clarified our introduction so it now reads: "Through biochemical reconstitutions, we demonstrate that mimicking multi-monoubiquitination of VASP reduces actin filament **side**-binding and bundling."

And yes, later on, we do show in Fig 4F that multi-monoubiquitination does not significantly change barbed end binding. However, we are not suggesting that the effects of monoubiquitination of VASP arise solely from this deficit in side binding. Although we do not see a change in barbed end binding, we do observe decreases in the elongation rate of mmUb-VASP compared to unmodified VASP in Fig 3C,E. In fact, the elongation rate of mmUb-VASP resembles the rate of actin alone---suggesting the ability of VASP to enhance actin monomer addition to the filament is impaired.

Lastly, as mentioned to Reviewer 1, we have performed bundling analysis on the timelapses from Figure 3D and have included this in Figure S2. These new analysis are described in the results as such:

"Actin filaments /bundles were traced to measure the mean intensity of actin along the structure to approximate bundling over time. We observed a significant increase in the mean fluorescence intensity of filaments with both unmodified VASP and mUb-K240 at ten minutes. However, no significant change was observed for filaments with mUb-K286 and mmUb-K240/K286, suggesting decreased bundling activity (**Fig S2**)."

Comment 2: I am a bit confused was to why the mass of the double ubiquitinated species in Figure 1E (bottom plot) isn't higher than the mono-ubiquitinated species (2nd and 3rd plot from the top)? The average mass of the multi ubiquitinated protein actually appears to be lower than what was seen for mono-ubiquitinated species. This is all the more perplexing since majority of VASP appears to be multi- ubiquitinated in the Coomassie gel shown in Fig. 1d. May be I am missing something here. Can authors clarify why this is the case?

Response: The molecular mass of VASP and ubiquitinated VASP was estimated by mass photometry (MP). Our primary objective in completing the experiment was to demonstrate a single population of the protein exists—corresponding to a tetrameric state. Although MP will calculate an approximate molecular weight, we observe an approximately 20-25% margin of error for each protein. While MP is sufficient to support the conclusion that mUb VASP and mmUb VASP still tetramerize, to limit any confusion, we have removed estimated molecular weights from Fig 1D. Further the text mentions: "No significant peak shifts were observed beyond the margin of error."

Comment 3: Fig. 3d. The contrast/intensity of the images could be improved (especially for those who read printed manuscripts). At first look, it appears that there are no filaments in the actin alone condition (top row). However, this is not the case. The filaments are just very dim.

Response: Thank you for this visual suggestion. Selection of image contrast/intensity in Fig 3D slightly complicated by the presence of dim single actin filaments and very bright VASP-mediated actin bundles. To circumvent this issue, we have replaced the two-color images shown in Figure 3D with only inverted LUT images of the actin channel to better visualize single actin filaments and highlight the changes in actin elongation rate at early movie timepoints.

We have relocated the two color images from these TIRF movies—showing later timepoints—to a new **Fig S2**, accompanying the bundling analysis of these images.

Comment 4: In Fig. 3D, I assume VASP is fluorescently labelled. Please mention this in the figure legend. It will also be helpful to expressly describe what the authors learn from the double-color TIRF experiments here. Can they use the data here to get an independent measure of VASP's side binding and bundling activities (see comment 1 above).

© 2023. Published by The Company of Biologists under the terms of the Creative Commons Attribution License (https://creativecommons.org/licenses/by/4.0/).

Response: The figure legend for 3D has been updated to specify the TAMRA-labeling of recombinant VASP.

Yes, as mentioned just above, we have created a new supplemental figure for this data to evaluate VASP bundling (**Fig S2**).

Comment 5: Authors (to their credit) note the conclusions from the pyrene assays in Fig. 3b don't entirely match their results of the TIRF assays. In pyrene assays, all 4 VASP conditions (unmodified VASP, mUB-240, mUB-286, mmUB-240/386) show higher polymerization than the condition with actin seeds. Due to addition of seeds, we can safely assume that the increase in pyrene signal is chiefly due to elongation and not nucleation. The authors "account this disparity to the higher sensitivity of the TIRF assays". More clarification is required here.

Response:

Thank you to the reviewer for pointing out the needed for more clarification. Seeded pyrene assays are a powerful tool to evaluate the elongation of actin filaments. However, this bulk assay examines filament elongation on a population level and we observed relatively large 95% confidence intervals from fitting the curves in **Fig 3C** in our attempts at calculating a B_{max} or maximum elongation rate. Alternatively, the TIRF assays allowed us to quantify the elongation rate of individual single actin filaments, providing more precise measurements at our concentration of interest (25 nM).

We have altered our text to now read: "Although we did not detect a significantly changed maximum elongation rate for mUb-K240 in the pyrene actin elongation assays, a large 95% confidence interval was calculated for these bulk measurements. In contrast, the ability to measure the elongation of single actin filaments in the TIRF assays may provide more accurate measurements."

Comment 6: Further in Fig. 3f, I am uncertain that any strong conclusions can be derived from the data presented here. At 1.25 nM, monoubiquitinated VASP is slightly faster than unmodified VASP; at 2.5 nM, monoubiquitinated VASP is >20% slower than unmodified VASP; and at 5 nM, monoubiquitinated VASP is almost as fast as unmodified VASP. So I am not sure if just based on 10 nM concentration, authors can conclude that ubiquitination impairs elongation by VASP.

Response:

In **Fig 3F**, we observe a statistically significant increase in the actin elongation rate in the presence of VASP at 2.5, 5, and 10 nM compared to actin alone. In contrast, mmUb did not significantly increase the actin elongation rate at any of the 4 concentrations (1.25 – 10 nM) tested in **Fig 3F**. 25 nM mmUb VASP also did not increase the elongation rate compared to actin alone in **Fig 3E**.

Although the reviewer qualitatively describes variation between VASP and mmUb at different concentrations, we do note that none of these comparisons are statistically significant. In contrast, we do observe a significant decrease in actin elongation rate between VASP and mmUb at 10 nM. This data also agrees with the significant decrease in actin elongation rate between VASP and mmUb at 25 nM in **Fig 3E**. Collectively, we believe the TIRF data displayed in **Fig 3E** and **3F** does highlight the inability of mmUb VASP to enhance actin filament elongation.

Comment 7: The role of profilin in VASP-mediated actin elongation as remained in debate. I am therefore unconvinced of authors' election to not test the effect of profilin in their experiments in Figure 3 and 4. I think it might help test if ubiquitin-modifications might influence VASP's activities in presence of profilin, especially since the effects in absence of profilin aren't striking.

Response:

Before addressing VASP and profilin interactions, we respectfully disagree ubiquitin modifications of VASP do not influence VASP activity, as appreciated also by both reviewers 1 and 3. We point to **Fig 3C** where we observe a change in the maximum actin assembly rate of unmodified VASP and

© 2023. Published by The Company of Biologists under the terms of the Creative Commons Attribution License (https://creativecommons.org/licenses/by/4.0/).

mmUb VASP. Although we cannot estimate the margin of error due to the incomplete curve fitting of mmUb (and therefore cannot perform statistical analysis), we investigate this further in Figure 3E. By TIRF microscopy, we estimate a median elongation rate of unmodified VASP at 13.2 subunits/sec and mmUb VASP at 7.084 subunits/sec (a statistically significant 46.3% reduction in elongation rate).

As VASP does contain a poly-proline binding site for profilin, we agree with Reviewer 2 that testing of profilin would be an excellent experiment. As eluded to by the reviewer, the published literature examining the utilization of profilin-actin by VASP—specifically in biochemical assays—is difficult to interpret. Barzik et al. 2005 suggests through seeded pyrene assays that profilin enhances the rate of VASP-mediated elongation compared to VASP alone. However only one experimental replicate is shown in the figure; an average of multiple replicates is not included, and thus lacks statistical analysis on this change. In contrast, Brühmann et al. 2017 concluded through TIRF assays that profilin-bound actin monomers do not enhance VASP mediated elongation.

We have included 2 new experiments in **Fig S3** to evaluate the ability of VASP and mmUb-VASP to utilize profilin actin in the absence or presence of capping protein.

In our hands and experimental conditions (approximately 50 nM ionic strength), we observe both VASP and mmUb-VASP are able to utilize profilin-actin, but profilin does not enhance the ability of either protein to elongate actin filaments over actin without profilin. These data are described in a new section of the results as such:

"N-terminal to the GAB domain, VASP also contains a poly-proline region that binds profilin-actin. Previously published in vitro experiments have mixed conclusions on the utilization of profilin-actin by Ena/VASP proteins due to differences in experimental design (Barzik et al., 2005; Hansen and Mullins, 2010; Breitsprecher et al., 2008; Brühmann et al., 2017). However, a recent cellular study suggests that Ena/VASP proteins do require profilin-actin for leading edge actin polymerization (Skruber et al., 2020). In our hands, we observed unmodified VASP (40 nM) was capable of utilizing profilin-actin. However, we did not observe an enhancement of actin elongation compared to VASP plus actin alone (**Fig S3A-C**). As seen before, mmUb-VASP failed to accelerate actin polymerization in the presence of actin alone, or in the presence of profilin. Because mmUb-VASP demonstrated a decreased ability to accelerate elongation, whether the mmUb-VASP could utilize profilin-actin was unclear. To circumvent this issue, we completed elongation assays in the presence of capping protein and profilin. We observed similar elongation rates of mmUb-VASP + actin + capping protein and mmUb-VASP + actin + capping protein + profilin. As a result, we do not detect any deficit of mmUb-VASP to utilize profilin-actin (**Fig S3D-F**).

Lastly, to determine if VASP ubiquitination impairs actin monomer binding, we performed fluorescence polarization experiments with 40 nM 488-labeled monomeric actin in low salt conditions that discourage polymerization. With increasing concentrations of unmodified VASP, we observed a dose-dependent increase in fluorescence polarization of monomeric actin, indicative of VASP binding. We observed an overlapping dose-dependent increase in fluorescence polarization with increasing concentrations of mmUb-K240/K286, suggesting the affinity of mmUb-K240/K286 to bind monomeric actin is similar to unmodified VASP (**Fig S3G**).

Comment 8: This is a minor point - in Fig. 4a, red spheres are supposed to denote capping protein. From the schematic, it appears two separate CP molecules can simultaneously bind barbed ends of an actin filament (2 red spheres occupy the barbed end of the actin filament). For sake of clarity, I suggest they adopt a different shape to denote CP such that only one molecule is bound at the barbed end at a time.

Response: We apologize for the confusion, as we had only intended to visualize the CP dimer. We have altered the cartoon accordingly.

Comment 9: "Conclusions", I assume they meant "Discussion". I found this section long and difficult to follow, I believe this section could be presented in a more concise manner. Also a more direct discussion about how their in vitro results explain (or not explain) their cellular results will be helpful.

© 2023. Published by The Company of Biologists under the terms of the Creative Commons Attribution License (https://creativecommons.org/licenses/by/4.0/).

Response: We have corrected the section title. Furthermore, as mentioned to Reviewer 1, we have removed several superfluous sections of the discussion. Lastly, we edited the discussion for brevity and added additional text to connect the in vitro and cellular experiments.

REVIEWER 3

Comment 1: The description of the mass spec and transfection experiments requires clarification. Fig. 1B is unclear. Can the authors indicate (color-code?) the Ub sites ID-ed in two different experiments? Were all the additional Ub sites detected in only one of the two samples or each of them ID-ed some additional unique sites?

Response:

We have updated our methods section and included additional experimental details regarding the proteomic mass spec experiments.

Yes, this is an excellent point. In **Fig 1B**, the 3 ubiquitination sites indicated with an asterisk were identified in 2 independent experiments. To discuss this in greater detail, we have added in **Fig S1A** to demonstrate the differences between our 2 biological replicates. Interestingly, our second replicate had a much higher expression rate of Myc-VASP. We also identified 13 ubiquitination sites in this replicate, compared to the 3 high confidence ubiquitination sites in replicate 1. We describe this in the results section as such:

"Following enzymatic digestion and analysis, we identified numerous lysine residues as ubiquitination sites on VASP by unbiased mass spectrometry and confirmed these by targeted analysis (**Fig 1B**). Despite multiple lysine residues identified, only three ubiquitination sites replicated across independent experiments and different enzymatic digestions. Of note, all of the additional ubiquitination sites were identified in the same experimental replicate, corresponding to a higher level of overexpression of Myc-VASP (**Fig S1A**)."

Comment 2: I am puzzled by so many potential sites of ubiquitination. Could it be an artifact of iodoacetamide treatment?

Response:

As discussed in Comment 1, we did see that our second replicate had many more ubiquitination sites than the first replicate, likely due to the higher expression of Myc-VASP in this experiment. For this reason, we selected only the two most abundant and replicated sites for study.

Our proteomic core is aware that iodoacetamide can cause di-carbamidomethylation of lysines, which has the same mass of the ubiquitin remnant digly on lysines; however, we do not think that this is the case here. Two replicates were analyzed - they were prepared for mass spectrometry and analyzed the exact same way and only the second replicate yielded a high degree of ubiquitin sites—likely due to the higher expression of Myc-VASP.

Comment 3: Exploration of the Ub modifications of the two most prominent sites is justified, however, the rationale for testing multi-Ub is not very clear to me. With so many potential Ub sites it's not obvious which mmUb species would be present in cells. Based on the data presented, ~10kDa shift in VASP protein population was consistent with a single Ub modification (potentially collective contribution of Ub on different single sites) but no prominent band corresponding to multi-Ub was detected. The authors should reconcile this.

Overall level of the VASP expression (endogenous+exdogenous) would be useful to know. Having "mock" condition (Lipofectamine only) shown side-by-side with the transfected samples would be helpful assuming that reliable VASP Ab are available. Is it possible that overexpression of VASP triggers the Ub at multiple sites? Some of these sites are regulatory but some of them could later target misfolded protein for degradation. Could lowering the expression level of exogenous VASP change the number of Ub detected?

© 2023. Published by The Company of Biologists under the terms of the Creative Commons Attribution License (https://creativecommons.org/licenses/by/4.0/).

Response:

We decided to test the multi-monoubiquitinated VASP mimics for a couple of reasons First is that the interpretation of molecular weights shifts by SDS-PAGE in general are complicated, but particularly so for VASP, and second because in neurons we have observed multiple discreet VASP-Ub bands. We now clarify this in the text as such:

"In neurons, we have observed two discrete VASP bands that co-migrate with ubiquitin, suggesting multi-monoubiquitination (Menon et al., 2015, Boyer et al., 2020). Studies utilizing ubiquitin mutants in HEK293 cells—identifying a single ubiquitinated VASP band—confirmed VASP mono- or multi-monoubiquitination (Boyer et al., 2020). As phosphorylation of VASP at Ser157 causes a disproportionally large apparent molecular weight shift (~4kDa) (Smolenski et al., 1998), we cannot accurately interpret the apparent molecular weight shift observed for VASP-Ub by SDS-PAGE as mono or multi-monoubiquitination. We created two VASP constructs with single cysteine mutations—VASP K240C and VASP K286C—within the cysteine-light VASP background construct to mimic two versions of monoubiquitinated VASP (mUb-K240 and mUb-K286). Based on the neuronal results suggesting multi-monoubiquitination (Menon et al., 2015, Boyer et al., 2020), we also created a double cysteine mutant VASP K240C, K286C to mimic a multi-monoubiquitinated version of VASP protein (mmUb-K240/K286) (**Fig 1D**)."

We have evaluated exogenous VASP levels in our proteomic inputs and normalized the levels of VASP to actin and have included this data in Fig S1A. We indeed see an approximately 3-fold increase in the level of over-expressed VASP in our second replicate. As the reviewer alludes, this corresponded to a higher number of identified ubiquitination sites compared to our first replicate (13 v. 3 sites, respectively). As a result, we do hypothesize some of these ubiquitination sites identified in the second replicate may be involved in the unfolded protein response/degradation pathway. We therefore prioritized investigating the abundant ubiquitination sites identified in the first proteomic replicate (as these sites were also identified in the 2nd replicate).

Comment 4: I think that the use of cross-linking was an elegant and justified approach. However, the authors should comment in the Results or Discussion on the potential limitations of using BMOE (which has a spacer arm length of ~8A) vs covalent bond formed between VASP and Ub under cellular conditions. Potentially, Ub attached via BMOE may have more motional freedom which (potentially) could contribute to the observed effects.

Response: We have added this text to the discussion under "**Study Limitations**". "In this work, we chose to mimic VASP ubiquitination with BMOE-mediated crosslinking—a sulfhydryl crosslinking approach with an 8 Å spacer arm. Although chemical ubiquitination can be facilitated with disulfide bond (estimated at 2 Å), disulfide bonds are sensitive to reduction by DTT. As actin polymerization assays are typically performed in the presence of 0.5 M DTT to reduce the labile cysteine present in actin, we chose to use the irreversible BMOE approach. As such, we must acknowledge this increased spacer length, which may alter the impact of ubiquitination on VASP activity."

Comment 5: Tables 2, 3 should be referred as Tables S1 and S2 in the main text. Additionally, I'd argue that fitting of the bulk pyrene assembly data might not yield true Kd values because of the combined contribution of actin nucleation, elongation, and (potentially) bundling to the signal. Additionally, the side-binding of VASP would change [VASP]free as actin polymerizes. Thus, I think it would be more accurate to use a different term such as Kd(app).

Response: We have labeled our tables as S1 and S2 and labeled this as K_D^{app}. In the legend fo the table we include the following statement: "Bmax and K_D^{app} calculated from actin pyrene assays. Because side-binding of VASP may change [VASP]free as actin polymerizes, and actin elongation and bundling may contribute to the bulk pyrene assembly data, we are reporting values as K_D^{app}."

Comment 6: Fig. 3D. I wonder if performing the analysis of the bundles' thickness using the movies which the team currently has would help better characterization the effects of Ub modifications.

Response: As requested by all three reviewers, we have quantified the mean filament fluorescence as a measurement of actin bundling in the TIRFM movies shown in Figure 3D. This data can be found in Figure S2. These new analysis are described in the results as such:

© 2023. Published by The Company of Biologists under the terms of the Creative Commons Attribution License (https://creativecommons.org/licenses/by/4.0/).

"Actin filaments /bundles were traced to measure the mean intensity of actin along the structure to approximate bundling over time. We observed a significant increase in the mean fluorescence intensity of filaments with both unmodified VASP and mUb-K240 at ten minutes. However, no significant change was observed for filaments with mUb-K286 and mmUb-K240/K286, suggesting decreased bundling activity (**Fig S2**)."

Second decision letter

MS ID#: JOCES/2023/261527

MS TITLE: Multi-monoubiquitination controls VASP-mediated actin dynamics

AUTHORS: Laura E McCormick, Cristian Suarez, Laura E Herring, Kevin S Cannon, David R Kovar, Nicholas G Brown, and Stephanie Gupton
ARTICLE TYPE: Research Article

It was good to see you at ASCB and am happy to tell you that your manuscript has been accepted for publication in Journal of Cell Science, pending standard ethics checks.

Reviewer 1

Advance summary and potential significance to field

The paper shows that ubiquitination of VASP inhibits its actin bundling and polymerization activity. The paper is significant because ubiquitination is a key mechanism of linking the signalling machinery to tuning actin cytoskeletal morphology.

Comments for the author

I am satisfied that the authors have answered my previous points and those of the other reviewers. The profilin experiments are a good addition, the quantification of the bundling adds to the manuscript and the discussion is much more focused and appropriate.

Reviewer 2

Advance summary and potential significance to field

Authors have addressed all my concerns and I have no further comments. I support publication of the manuscript as is.

Comments for the author

Authors have addressed all my concerns and I have no further comments. I support publication of the manuscript as is.

Reviewer 3

Advance summary and potential significance to field

This study presents novel mechanistic insights related to VASP regulation by ubiquitination (Ub).

Comments for the author

© 2023. Published by The Company of Biologists under the terms of the Creative Commons Attribution License (https://creativecommons.org/licenses/by/4.0/).

The authors addressed my comments in the revised version of the manuscript. I recommend this work for publication in Journal of Cell Science.

© 2023. Published by The Company of Biologists under the terms of the Creative Commons Attribution License (https://creativecommons.org/licenses/by/4.0/).
